# Differentiated Zn(II) binding affinities in animal, plant, and bacterial metallothioneins define their zinc buffering capacity at physiological pZn

**DOI:** 10.1093/mtomcs/mfad061

**Published:** 2023-10-06

**Authors:** Karolina Mosna, Kinga Jurczak, Artur Krężel

**Affiliations:** Department of Chemical Biology, Faculty of Biotechnology, University of Wrocław, Joliot-Curie 14a, 50-383 Wrocław, Poland; Department of Chemical Biology, Faculty of Biotechnology, University of Wrocław, Joliot-Curie 14a, 50-383 Wrocław, Poland; Department of Chemical Biology, Faculty of Biotechnology, University of Wrocław, Joliot-Curie 14a, 50-383 Wrocław, Poland

**Keywords:** free zinc, metal buffer, protein folding, zinc speciation, zinc transfer, ZnAF-2F

## Abstract

Metallothioneins (MTs) are small, Cys-rich proteins present in various but not all organisms, from bacteria to humans. They participate in zinc and copper metabolism, toxic metals detoxification, and protection against reactive species. Structurally, they contain one or multiple domains, capable of binding a variable number of metal ions. For experimental convenience, biochemical characterization of MTs is mainly performed on Cd(II)-loaded proteins, frequently omitting or limiting Zn(II) binding features and related functions. Here, by choosing 10 MTs with relatively well-characterized structures from animals, plants, and bacteria, we focused on poorly investigated Zn(II)-to-protein affinities, stability–structure relations, and the speciation of individual complexes. For that purpose, MTs were characterized in terms of stoichiometry, pH-dependent Zn(II) binding, and competition with chromogenic and fluorescent probes. To shed more light on protein folding and its relation with Zn(II) affinity, reactivity of variously Zn(II)-loaded MTs was studied by (5,5ʹ-dithiobis(2-nitrobenzoic acid) oxidation in the presence of mild chelators. The results show that animal and plant MTs, despite their architectural differences, demonstrate the same affinities to Zn(II), varying from nano- to low picomolar range. Bacterial MTs bind Zn(II) more tightly but, importantly, with different affinities from low picomolar to low femtomolar range. The presence of weak, moderate, and tight zinc sites is related to the folding mechanisms and internal electrostatic interactions. Differentiated affinities of all MTs define their zinc buffering capacity required for Zn(II) donation and acceptance at various free Zn(II) concentrations (pZn levels). The data demonstrate critical roles of individual Zn(II)-depleted MT species in zinc buffering processes.

## Graphical Abstract

**Graphical Abstract:**
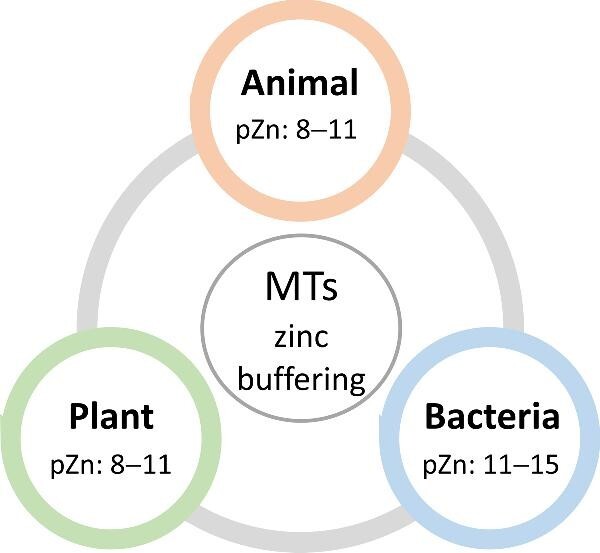
Zinc buffering properties of animal, plant, and bacterial metallotioneins.

## Introduction

Metallothioneins (MTs) are small, Cys-rich proteins, whose common feature is the binding of essential metal ions such as Zn(II), Cu(I), and various toxic metal ions.^1^ The first mammalian MT was discovered in the horse's kidney, and in the following years, its isolation from natural mammalian sources and biophysical characterization were improved.^2–4^ Over time, it turned out that MTs are found not only in mammals, but also in other taxonomic groups, including aquatic organisms such as fishes, bivalves, zooplankton, and crustaceans.^5–8^ The first prokaryotic bacterial MT in *Synechococcus elongatus* was found in the 1970s.^9^ Further bacterial MTs have been discovered in *Pseudomonas* sp., *Synechococcus* sp., and *Anabaena* sp. using gene DNA sequence analysis.^10–12^ In 2008, the first MT from the pathogenic bacterium *Mycobacterium tuberculosis* was discovered; it has a sequence motif that does not appear in other bacterial MTs and is considered copper MT.^13^ The first plant MT containing Zn(II), named as wheat-germ Ec protein, was isolated in 1987 from wheat embryos.^14,15^ The discovery of plant MTs coincided with the discovery of Cys-rich peptides also capable of binding heavy ions named phytochelatins (PCs).^16^ Despite their comparable metal binding properties, they cannot be considered as MTs since they are produced in noncoding enzymatic synthesis in contrast to MTs, whose sequences are encoded in DNA.^17^ Further findings of plant MTs were not as dynamic as in the case of animals, as there were problems with the isolation of new plant MTs.^18^ The ability to produce recombinant proteins in a variety of expression systems has again accelerated MT research.^19,20^ Nowadays, MTs are perceived as a diverse group of small proteins containing one or multiple domains, able to bind various metal ions in metal clusters by Cys and His residues in various geometries.

Over the years, the majority of biochemical research on various MT was mainly based on their interaction with Cd(II).^1^ This element was found in the first discovered MT.^2^ It has also been proven that exposure of the organism to Cd(II) and Zn(II) ions can lead to overexpression of MT, which facilitated the isolation and identification of MTs from the new organisms.^21–23^ The additional advantage of research with cadmium is the spectroscopic properties of metal complexes, which can be investigated by many techniques such as ultraviolet-visible spectroscopy (UV–vis), circular dicroism/magnetic circular dicroism spectroscopy (CD/MCD), nuclear magnetic resonance spectroscopy (NMR), or X-ray spectroscopies in contrast to much more spectroscopically silent Zn(II) complexes.^1,24–26^ Although Zn(II) and Cd(II) are chemically similar, they demonstrate different preferences for MT domains and folding mechanisms.^27–29^ Another reason for this situation was the initial assumption of the principal role of MTs as toxic metal ions detoxifying proteins.^30^ Moreover, the time of MTs’ discoveries overlapped with the time when it was proved that exposure to cadmium can cause severe diseases, including carcinogenesis.^31,32^ Studies with Zn(II) have shown that apart from their detoxifying functions, MTs primarily act as reservoirs and a buffer system of these ions.^33,34^ As pointed out by Bremner and Davis, the binding of Cd(II) or Hg(II) to MTs may be an accidental consequence of the similarity of these ions and difference in the affinities or may happen under stressed conditions.^33^ Currently there is no single criterion established to determine that one MT protein is Zn(II) or perhaps Cu(I) or Cd(II) specific. What is currently known is that animal (not marine or soil organism) MTs are proteins involved in the metabolism of zinc and copper, and in the case of exposure to other thiophilic metals, their native metals are simply substituted due to the laws of chemistry.^35,36^ It should be, however, remembered that there are a plethora of proteins (mostly in bacteria) that bind toxic metals, e.g. Cd(II), such as CadC family, corresponding efflux pumps and systems, and carbonic anhydrase from *Thalassiosira weissflogii* diatoms where the catalytic ion is Cd(II), but it can be replaced by Zn(II).^37,38^ Therefore, toxic metals binding to MTs of bacterial, plant or marine organisms more likely occurs due to their environmental exposition.

Among all physicochemical parameters of metalloproteins that determine their biophysical properties and functions, metal(s) binding affinity is one of the most important. In the case of zinc proteins, the affinity of Zn(II) toward protein may vary by several orders of magnitude from the micro- to low femtomolar range depending on their extra- or intracellular localization, ligand composition, protein architecture, or mechanism of zinc site folding.^39–42^ Structural and enzymatic zinc sites are the most stable, and high Zn(II) binding affinity ensures constant saturation under cellular conditions, which varies among domains of life.^39–41,43^ Regulatory zinc proteins bind Zn(II) transiently, and because of that fact the binding affinity must be moderate and occur in the range of free Zn(II), whose concentrations depends on the whole zinc homeostatic machinery and total metal concentration.^1,39,40,44^ The level of free Zn(II) ([Zn(II)]_free_ presented in the text also as logarithmic value pZn = −log[Zn(II)]_free_) in animal and plant cells is similar, estimated to be in the pico-/nanomolar range, while in bacteria it is lower, in the pico- to femtomolar range.^45–47^ In mammals, MTs are recognized as regulatory proteins that constitute part of the zinc homeostatic machinery. It has been shown in the example of mammalian MT2 that it binds seven Zn(II) ions (in Zn_7_MT form) with various affinities from low picomolar to nanomolar range.^48^ The differentiation of particular zinc sites’ affinities has significant consequence for the speciation of that protein, which under cytosolic subnanomolar free Zn(II) concentration or its natural fluctuations is not fully saturated by Zn(II) and is present as the partially metalated species Zn_4-6_MT. It ensures zinc buffering properties of this protein and maintains the free Zn(II) concentration in the pico-/nanomolar range. So, if the free Zn(II) concentration in animal and plant cells is similar (as the data show), there arises a question about the overall role of MTs. Do their functions vary depending on the species? Do they play a role in zinc buffering under various cellular conditions? Finally, how do bacterial MTs differ in thermodynamic terms from eukaryotic MTs, and what molecular factors could explain their differentiation?

Here, we answer at least some of the questions by providing detailed characterization of Zn(II) complexes with MTs from animal, plant, and bacteria. Our main interest is oriented on Zn(II) binding affinities of previously studied MTs. It should be underlined that stability data of Zn(II)-MTs systems be obtained so far under various conditions using different methodologies and show mainly, besides few exceptions, a single affinity of all zinc sites, which simplified the system to apo- and fully loaded forms only.^1,49^ Therefore, the aim of this study was also producing a coherent set of affinity data of all zinc sites in particular MTs determined by the same methodology and under the same conditions, in order for allowing direct comparisons. We believe this report will shed more light on the whole family of MTs and their biochemical characterization.

## Results and discussion

### Selection of MTs

**Fig. 1:**
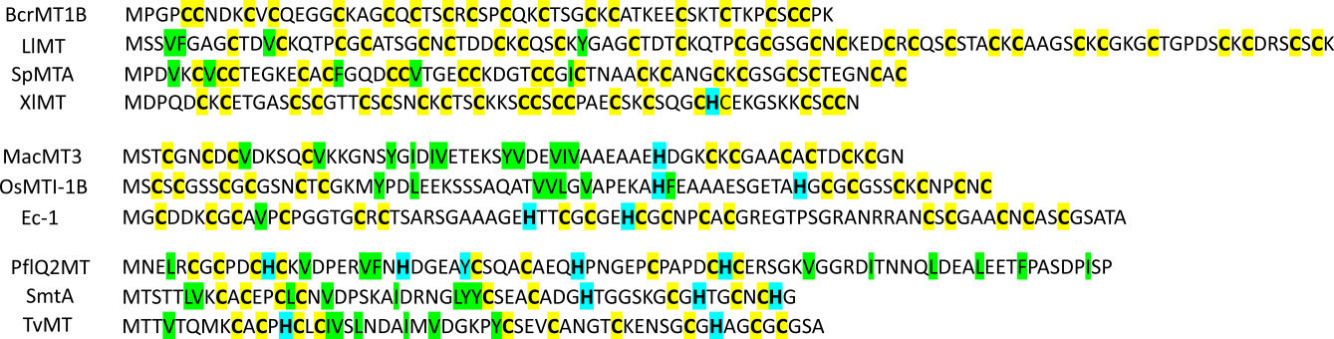
Amino acid sequences of MTs used in this study. Top, middle, and bottom panels correspond to animal, plant and bacterial MTs, respectively. Yellow, blue, and green colors highlight cysteine, histidine, and hydrophobic amino acid residues, respectively.

Because MTs are present in various organisms, which demonstrate fundamental differences in functioning, they show different metabolism of essential metal ions. To date MTs have been found in animals, plants, fungi and bacteria. However, only a few of them have been characterized in terms of their biophysical properties, especially Zn(II) binding. Although average Zn(II) binding constants were determined for some MTs over the years, only in the case of mammalian MTs step binding constants of all seven zinc sites were determined,^50–52^ indicating the importance of individual partially and fully Zn(II)-loaded species (Zn_7-_*_x_*MT) in zinc buffering processes.^29,48,49^ Even though organisms producing MTs are distinctly different, the sequences of these proteins share common features. Therefore, it is worth examining whether or not they demonstrate similar or substantially different Zn(II) binding properties, similar to mammalian counterparts. Hence, the main goal of this study was to examine whether bacterial, animal (other than mammalian), and plant MTs exhibit unique zinc buffering properties capable of buffering the free Zn(II) concentrations found in these organisms. By zinc buffering properties, we understand Zn(II) binding or its dissociation from Zn(II)-loaded and depleted species occurring under physiological pZn, demonstrating different chemical and structural features. The capacity of MT-based zinc buffer is related to the number of zinc sites and differentiation of their affinities for Zn(II).^1,49^ Examination of these features would shed light on the role of MTs in zinc metabolism within the organisms. Here, we selected three to four organisms (from animals, plants, and bacteria), for which MTs have been relatively well or at least partially characterized in terms of biophysical and metal binding properties (see preceding and following text). The selected animal organisms and their MTs are *Callinectes sapidus* (blue crab, BcrMT1B), *Littorina littorea* (common periwinkle, LlMT), *Strongylocentrotus purpuratus* (purple sea urchin, SpMTA), and *Xenopus laevis* (African clawed frog XlMT). The plant organisms are *Musa acuminata* (banana, MacMT3), *Oryza sativa japonica*(Japonica rice, OsMTI-1B), and *Tritium aestivum* (common wheat, Ec-1). The bacteria are *Pseudomonas fluorescens* Q2-87(PflQ2MT), *S. elongatus* PCC 7942(SmtA), and *Thermosynechococcus vulcanus* (TvMT). Amino acid sequences of all MTs are presented in Fig. 1. It highlights cysteine and histidine residues that are capable of Zn(II) binding. Histidine residues are the most common in bacterial and plant MTs and remain unique in animal MTs. The same is true for hydrophobic residues. Although selected proteins were partially investigated, it should be mentioned that TvMT is characterized here for Zn(II) binding properties for the first time.

### Production and purification of metal-free and Zn(II)-loaded MT forms

In this study two types of MTs were used, metal-free forms called apo-MT or thionein and a fully Zn(II)-loaded holo-form (MT) obtained under specific conditions (see section “Experimental procedures”). Due to the high content of Cys residues, produced proteins were reduced before purification and kept reduced at all stages to obtain homogeneous material (reduced and fully loaded under used conditions). The IMPACT system is highly useful for that purpose because self-cleavage of the fusion proteins (containing chitin-binding protein, an intein fragment and MT) occurs on the chitin column in the presence of 0.1 M DTT (DL-dithiothreitol), which catalyses the self-cleavage reaction and reduces disulfide bridges if present. After this process reduced proteins were acidified to pH ∼2.5 to promote dissociation of metal ions bound in *E. coli*.^53^ Moreover, low pH protects Cys residues against oxidation during size-exclusion chromatography (SEC) purification and short-term storage. Collected fractions were either used directly for experiments or concentrated and used for reconstitution (see following text). The concentrations of freshly prepared and reduced apo-MTs were determined using the DTNB (5,5ʹ-dithiobis(2-nitrobenzoic acid) assay.^48,54^ In order to check the identity of obtained metal-free proteins, their molecular masses were determined by electrospray ionization–mass spectrometry (ESI–MS) and the recorded masses as well as calculated masses are presented in Supplementary Table S2. All masses are convergent, indicating correct identity and the reduced state. Table 1 presents molar absorption coefficients of all apo-MTs determined at 215 and 220 nm, in 10 mM HCl since it was used in chromatography as a mobile phase.

**Table 1.** Molar absorption coefficients of apo- and Zn(II)-loaded forms of investigated metallothioneins^a^

	apo-MT		
Metallothionein	ε_215_ (M^−1^· cm^−1^)	ε_220_ (M^−1^· cm^−1^)	Zn(II)-MT ε_220_ (M^−1^· cm^−1^)
BcrMT1B	88 500 ± 200	56 900 ± 150	145 000 ± 2 000
LlMT	126 600 ± 200	82 800 ± 200	228 000 ± 400
SpMTA	80 200 ± 200	50 800 ± 300	161 000 ± 2000
XlMT	82 450 ± 70	53 940 ± 100	164 000 ± 1000
MacMT3	82 000 ± 2000	61 000 ± 2000	107 000 ± 1 000
OsMTI-1B	115 000 ± 1000	79 500 ± 700	133 800 ± 100
Ec-1	90 700 ± 600	58 700 ± 500	161 000 ± 2000
PflQ2MT	150 700 ± 1000	102 400 ± 1000	148 000 ± 7000
SmtA	82 100 ± 300	59 100 ± 200	98 000 ± 1000
TvMT	78 280 ± 50	54 600 ± 200	110 000 ± 700

^a^Data are shown as means of *n* = 3 independent experiments + SD.

Homogeneous Zn(II)-loaded proteins were obtained from pure apo-MT forms by their ZnSO_4_ saturation.^53,55^ To avoid oxidation of apo-MTs, they were mixed with TCEP (tris(2-carboxyethyl)phosphine hydrochloride) and then with ZnSO_4_ in acidic conditions. ZnSO_4_ excess over the expected protein load was 15–20%. The pH adjustment to the value 8.6 causes immediate full Zn(II) protein saturation. The excess of ZnSO_4_ was removed during the second SEC purification step, which was run at pH 8.6. This pH value avoids Zn(II) dissociation during this purification and efficiently protects zinc proteins against oxidation since they are more prone to oxidation than their cadmium counterparts due to lower metal affinity.^1^ It should be mentioned that TCEP was used in this study as a weakly Zn(II) binding reducing agent in contrast to dithiol agents such as DTT or DTBA (dithiobutylamine).^56,57^ Homogeneous Zn(II)-MTs were used immediately for the experiments or stored at −80°C. Molar absorption coefficients at 220 nm of all Zn(II)-loaded MTs determined in 50 mM borate buffer, 100 mM NaClO_4_,pH 7.4 are presented in Table 1. They were calculated using protein concentrations determined by spectroscopic assays and inductively coupled plasma (ICP) measurements. The coefficients were used later for the determination of MT concentrations from new preparations.

### Analysis of Zn(II) and thiolate contents

Freshly reconstituted MTs were analysed for Zn(II) and thiolate concentrations using several differentiated approaches. In the first one Zn(II) concentrations in MT samples were quantified by a chromophoric chelating probe, PAR (4-(2-pyridylazo)resorcinol). Its Zn(II) binding affinity is not high enough to remove all Zn(II) ions from Zn(II)-loaded proteins (log*K*_12_ of ZnH*_x_*(PAR)_2_ is 12.15).^58^ Therefore, metal concentration was measured after the addition of DTNB, which was shown to accelerate Zn(II) transfer due to thiolate oxidation in mammalian MTs.^59^ Zn(II) binding to PAR was monitored in a time-dependent mode to ensure that metal quantification was performed at the end of the reaction. Thiolate concentrations were determined by DTNB assay in the presence of ethylenediaminetetraacetic acid (EDTA) to accelerate oxidation reaction, and monitored over time. Both assays were run to reach absorbance constant values. It should be mentioned, however, that in the case of PflQ2MT and TvMT spectroscopic assays failed due to the very long time necessary for total protein oxidation and Zn(II) dissociation being beyond assay accuracy. Table 2 presents Zn(II) equivalents in investigated MTs counted per protein concentration determined from both spectroscopic assays. For PflQ2MT and TvMT it was impossible to compare both methods.

**Table 2.** Analysis of Zn(II) or Cd(II)-to-protein ratios based on spectroscopic and ICP analysis of investigated metallothioneins^a^

Metallothionein	Zn(II) mol. eq. PAR and DTNB assay^b^	Zn(II) mol. eq. ICP^c^	Zn(II)/sulfur ICP	Zn(II) mol. eq. MS^d^	Zn(II) mol. eq. UV titration^e^	Cd(II) mol. eq. UV titration^e^
BcrMT1B	5.4	6.6	5.5	6	5.5	6
LlMT	9	8.6	9.7	9	8	9
SpMTA	6.8	7.9	7.8	7	6	7
XlMT	7	7.7	7.1	7	5.5	7
MacMT3	2.6	2.5	2.6	3	3.5	4
OsMTI-1B	3.6	3.6	3.8	4	3	4
Ec-1	5.6	6.4	6.2	6	6	6
PflQ2MT	*n.d.*	3.5	3.8	3	3	4
SmtA	3.8	4.5	4.1	4	3	4
TvMT	*n.d.*	4.7	4.7	4	3.5	4

^a^ *n.d.* denotes not determined due to technical problems.

^b^Zn(II) concentration determined by PAR per protein concentration calculated using DTNB assay.

^c^Zn(II) concentration determined by ICP per protein concentration calculated using DTNB assay.

^d^The value of the highest intensity species.

^e^Values averaged based on various wavelengths.

Independently to the spectroscopic analysis, zinc and sulfur contents in MT samples were analysed using ICP–AES (inductively coupled plasma-atomic emission spectroscopy). Metal equivalents obtained from spectroscopic and ICP–AES are convergent as presented in Table 2. However, in the case of LlMT, SpMTA, and TvMTZn(II) equivalents and Zn/S ratios obtained from ICP measurements are higher than indicated by spectroscopic studies. Similar results were obtained for LlMT in the previous study by Palacios *et al*.^20^ This can be explained by partial protein precipitation during wet mineralization since precipitates are not available in contrast to released metal ions in low pH which are available for ICP measurements.

Another method used for the investigation of Zn(II)-MT stoichiometries and Zn(II) content in recombinant proteins was MS. For that purpose, purified and concentrated samples were taken for ESI–MS analysis in native conditions including use of 50 mM ammonium acetate solution, pH 7.4. Recorded mass spectra indicate gas phase speciation of Zn(II)-loaded MTs under used conditions (Supplementary Fig. S1; Supplementary Table S3). It should be noted that signals of Zn(II)-MT complexes should be taken qualitatively rather than quantitatively when measured by MS, which has been shown by numerous studies.^28,49,60^ The maximum Zn(II) load for BcrMT1B, LlMT, SpMTA, XlMT, MacMT3, OsMTI-1B, and Ec-1 was 6, 9, 7, 7, 3, 4, and 6, respectively.^20,61^^–^^64^ These values are convergent with those reported for these proteins in the literature, besides OsMTI-1B, for which binding of only two Zn(II) ions was reported, but for BcrMT and XlMT there are no data on the interactions with Zn(II).^65^ For all earlier mentioned besides XlMT partially Zn(II)-depleted species were also observed (Supplementary Fig. S1; Supplementary Table S3), which may suggest lower complex stability in the gas phase due to high dynamic structure or lower Zn(II)-to-protein affinity.^66^ However, stability data presented in the following text show that this protein demonstrates similar properties to all those mentioned. Regarding Zn(II)-loaded PflQ2MT, SmtA and TvMT, their spectra demonstrate exclusively one signal (for PflQ2MT there is slight broadening), indicating much more stable structure in the gas phase compared to animal and plant MTs’ spectra.^66^ The number of bound Zn(II) ions is 3 and 4, respectively, which confirms results reported previously.^67,68^ However, for TvMT, BcrMT1B, and XlMT it is the first information regarding Zn(II)-to-MT stoichiometry.

### UV–vis and CD spectroscopic studies of Zn(II) and Cd(II) complexation by MTs

**Fig. 2:**
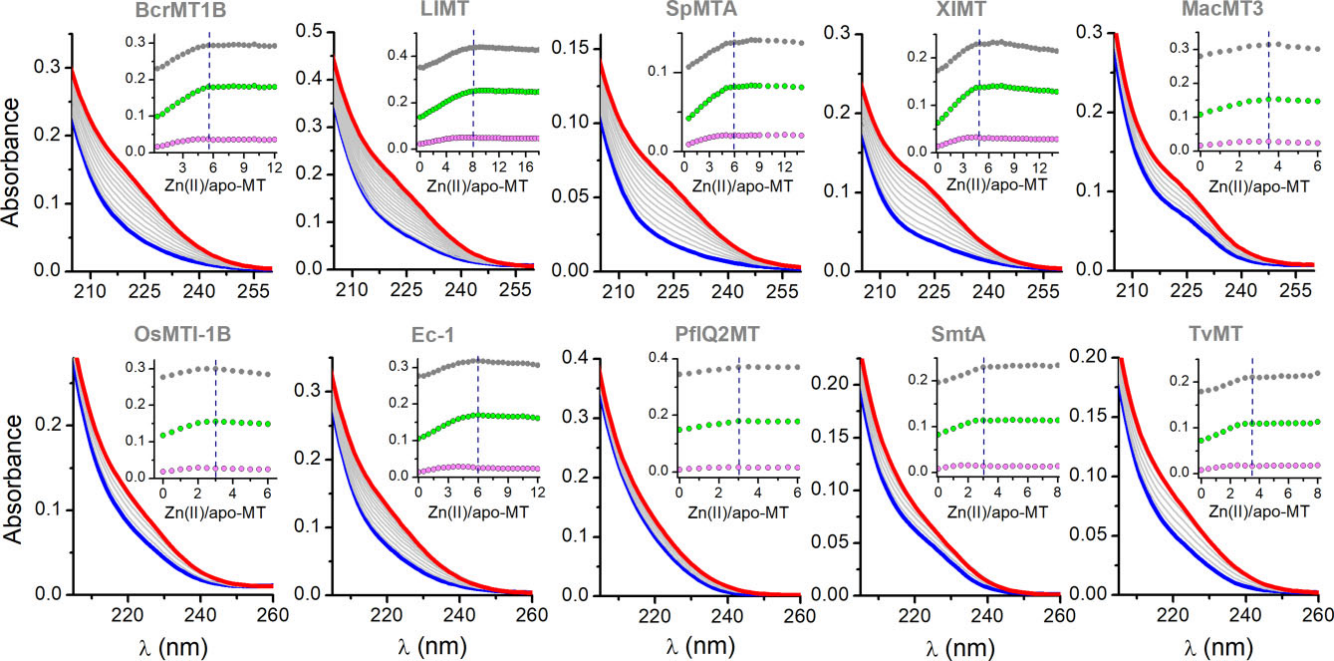
Spectrophotometric titrations of metal-free MTs with Zn(II) in UV range. Spectra were recorded for 1 µM apo-MT (blue lines) titrated with ZnSO_4_. Zn(II)-saturated MTs are shown as red lines. Spectra were recorded in 50 mM borate buffer, 100 mM NaClO_4_, 45–135 µM TCEP, pH 7.4. Insets demonstrate absorbance increase at 205 (gray circles), 215 (green circles), and 240 nm (magenta circles). Dashed lines indicate saturation of the signals.

Although some spectroscopic data of Zn(II) and Cd(II) interactions with investigated proteins are present in the literature, here we performed full characterization of all selected proteins under the same conditions to compare them in detail. Figure 2 presents UV-range monitored titrations of all apo-MTs with Zn(II), while Cd(II) titrations are presented in Supplementary Fig. S2. Zn(II) binding to MTs is accompanied by the formation of a band at ∼220 nm, and for Cd(II) a band at ∼240 nm. Since observable ligand-to-metal charge transfer (LMCT) bands are observable as shoulders (mostly for Zn(II)), their maximum wavelengths are readable in differential spectra presented in Supplementary Figs. S3 and S4 for Zn(II) and Cd(II), respectively. Zn(II) binding isotherms plotted at maximum wavelengths (insets in Fig. 2) are linear at the first part of the titrations and more rounded when close to titration endpoints, which is in contrast to Cd(II) titrations (insets of Supplementary Fig. S2). The latter are more linear throughout titrations and the saturation points are sharper compared to Zn(II) titrations. Endpoints of Zn(II) titrations occur at slightly lower M/apo-MT values (M indicates Zn(II) or Cd(II)) than for Cd(II)-based experiments. All these values are presented in Table 2 and provide information regarding the stoichiometry of fully loaded complexes. For instance, Zn(II) titrations for BcrMT1B, LlMT, and SpMTA suggest the binding of 5, 8, or 6 ions, while Cd(II) titrations clearly show the binding of 6, 9, and 7 metal ions. It is similar for the rest of the MTs; however, not in all cases is the difference between the two elements so clear and exactly 1 mol. eq. (Table 2). The observable difference between metal ions comes from different mechanisms of protein folding and it was observed in the previous study.^28,69^ Cd(II) ion besides being a softer Lewis acid also has a higher tendency to form Cd*_x_*S*_y_* clusters compared to Zn(II), binding of which is more entropy driven and occurs along the MT molecule. Our recent combined study based on MS and molecular dynamics showed that Zn(II) ions in human MT2 bind to both domains at the first stage of the metalation process while clustering occurs in the other steps, which results in a linear and then rounded shape of absorbance increase since the intensity of LMCT is higher for terminal (M–S) than for bridging (M–S–M) bonds.^48,70^ In contrast, more cooperative Cd(II) complexation results in comparable absorbance increases per binding event, which is reflected in more linear titrations.^28^ Regardless of some differences between elements in UV titrations, PAR/DTNB spectroscopic assays and ICP–AES measurements show binding of the same number of Zn(II) ions as is observable for UV–vis Cd(II) titrations, indicating convergence of these approaches. Although literature data demonstrate Zn(II) complexation by Ec-1, MacMT3, LlMT, SpMTA, PflQ2MT, and SmtA, UV–vis Zn(II) titrations were performed only for MacMT3 and PflQ2MT, which indicated the binding of 4 Zn(II) mol. eq.^20,62^^–^^64,67,71^^–^^73^ It should be underlined, however, that metal-free MacMT3 when titrated with Zn(II) is capable of binding of 4 Zn(II) mol. eq. at metal excess. Nevertheless, reconstituted protein from bacterial expression indicates Zn_3_MacMT3 stoichiometry, which was also observed in the previously isolated protein.^64,71^ It demonstrates that the fourth Zn(II) ion is bound loosely and this event is observable only at metal excess, which occurs only in Zn(II)-to-protein titrations. The fourth Cd(II) ion binds to MacMT3 with comparable affinity to the remaining three ions.

**Fig. 3:**
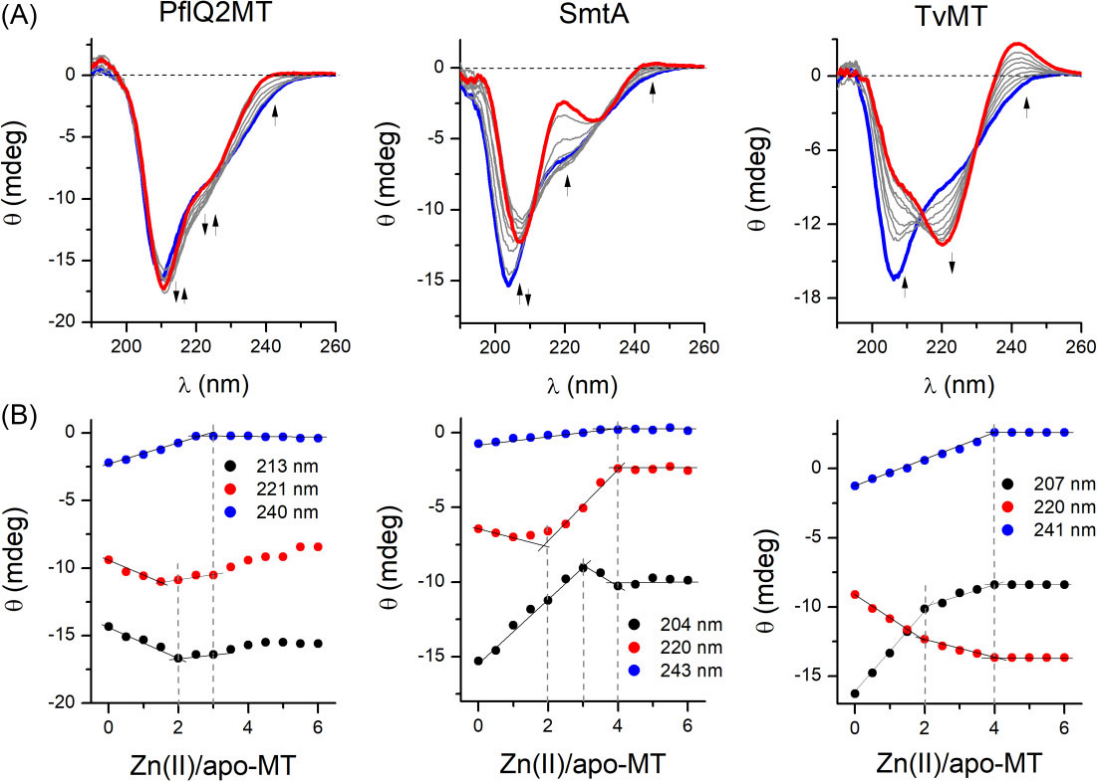
CD-monitored titrations of metal-free bacterial MTs with Zn(II). (A) CD spectra of 20 µM apo-MT titrated with ZnSO_4_ in 10 mM Tris-HCl, 100 mM NaClO_4_, 360 µM (PflQ2MT, SmtA) or 400 µM (TvMT) TCEP, pH 7.4. Red and blue lines demonstrate spectra of apo-MTs and Zn(II)-saturated MTs. Arrows indicate signal changes. (B) Ellipticities at selected wavelengths as a function of increasing Zn(II)-to-apo-MT ratios.

Zn(II) and Cd(II) binding to MTs was also analysed by circular dichroism (CD) spectroscopy. It should be mentioned, however, that Zn(II) binding to highly dynamic MTs does not result in intensive CD signals and that method is very often used for studying Cd(II)-to-MT binding, which requires a much lower protein concentration.^24^ Therefore, here CD study was applied for only more rigid MTs, which form well-folded Zn(II) complexes, namely for PflQ2MT, SmtA, and TvMT. Figure 3 and Supplementary Fig. S5 present CD titrations of metal-free MTs with Zn(II) and Cd(II), respectively. PflQ2MT as the only bacterial protein investigated in this study does not form highly intensive spectra with Zn(II) (Fig. 3A). However, the ellipticity change at 240 nm indicates complex formation at 3 Zn(II) mol. eq. (Zn_3_PflQ2MT) while changes at 213 and 221 nm additionally show the formation of Zn_2_PflQ2MT species (Fig. 3B). Cd(II) spectra of PflQ2MT are substantially different (Supplementary Fig. S5A), indicating formation of a more helical structure upon Cd(II) saturation. This process occurs up to 3 Cd(II) mol. eq. (Supplementary Fig. S5B), while the addition of one more Cd(II) mol. eq. results in a subtle but clear change of CD signals, especially visible at 253 nm, where a new band is formed. The difference between Zn(II) and Cd(II) complexes was reported previously by Habjanič *et al*.^67^ SmtA folding is nicely observable for metal ion complexation. The ellipticity change at 243 nm (Fig. 3A) indicates formation of the Zn(II) complex with maximum 4 mol. eq. while changes at 204 and 220 nm show formation of Zn_2_SmtA and Zn_3_SmtA species. Cd(II) metalation results in the same stoichiometries of formed complexes as Zn(II) (Supplementary Fig. S5). These observations are convergent with previously obtained results; TvMT forms complexes very similar to SmtA due to the similar amino acid sequence.^68^ Interestingly, very intensive signals of Cd(II)-TvMT spectra with at 243 and 267 (Supplementary Fig. S5) indicate a strong LMCT contribution. As pointed out earlier, CD-based characterization of TvMT complexes was performed for the first time, indicating that its Zn(II) and Cd(II) binding abilities are highly similar to SmtA.

### Zn(II) metalation mechanism by oxidation of MTs with DTNB

**Fig. 4:**
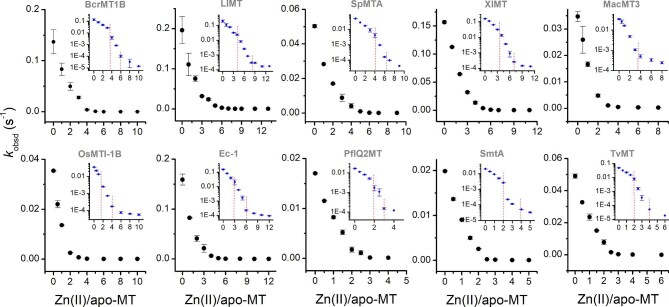
Titrations of Zn(II) to metal-free MTs monitored by protein oxidation with DTNB. A total of 1 µM of a particular apo-MT was incubated with increasing ZnSO_4_ equivalents and probed for the oxidation rate with 1 µM DTNB in 50 mM Na^+^-HEPES buffer, 100 mM NaCl, pH 7.4. Increased TNB^−^ product concentration over time was fitted to the first order kinetics equation to obtained *k*_obsd_ values used for preparation of graphs. Red dashed lines indicate characteristic endpoints (if observable). Data are shown as means of *n* = 2 independent experiments + SD.

To shed more light on the mechanism of Zn(II) complexation to investigated MTs and binding sites’ differentiation regarding their affinity, we used the thiol(ate) reactivity test, which is based on rapid free thiol oxidation by DTNB at equimolar concentration of protein and an oxidant, and significantly slower reactivity of thiolates (Cys bound to Zn(II)).^48,74^ For that purpose, apo-MTs and samples with increasing Zn(II)-to-protein molar ratios were probed for TNB^−^appearance velocities. Figure 4 shows the relations between pseudo-first order kinetic constants (*k*_obsd_) of TNB^−^ formation upon protein oxidation and the number of Zn(II) equivalents added to the proteins. All MTs demonstrate similar trends showing very effective reduction of oxidation kinetics within added ZnSO_4_. Efficient decrease of *k*_obsd_ occurs up to characteristic Zn(II)-to-protein ratios (first titration endpoint) followed by a slower decrease in the trend. This slower trend can still be monitored using logarithmic plots as presented in insets of Fig. 4. For instance, in the case of BcrMT1B, an efficient drop of *k*_obsd_ occurs up to 4 Zn(II) mol. eq., indicating a dramatic decrease of protein reactivity towards DTNB in this region. The addition of another ∼3 Zn(II) mol. eq. results in an additional reactivity decrease forming the second titration endpoint, above which almost no change of oxidation velocity is observable. Interestingly, the first titration endpoints do not correlate with the total number of Zn(II) mol. eq. bound to the protein as determined by spectroscopic assays or ICP. The total number of Zn(II) bound to protein is instead visible at the second endpoints of the titration. An identical scenario was previously observed for human MT2, the metalation process of which has been explored in detail using many different methods and approaches.^28,48,49,59,69^ In that case four Zn(II) ions were found to bind to both α- and β-domains in such a way that large a number of Cys residues are involved in Zn(II) coordination. This fact causes a dramatic decrease of DTNB oxidation velocity, indicating a significantly lower number of available free thiols due to the metalation mechanism and protein structure formation, which additionally acts as a limiting factor of MTs’ reactivity. Similarly to human MT2, proteins investigated in this study show a similar trend in the metalation mechanism. Data presented in Fig. 4 indicate that in the first step of protein folding 4 (BcrMT1B), 4 (LlMT), 4 (SpMTA), 3–4 (XlMT), 2 (MacMT3), 2 (OsMTI-1B), 3–4 (Ec-1), 2 (PflQ2MT), 2 (SmtA), and 2 (TvMT) Zn(II) ions bind in such a way as to coordinate the maximum possible number of Cys residues in MTs, which results in a dramatic decrease of the reactivity of these partially Zn(II)-loaded species. The second stage requires the binding of additional Zn(II) ions up to a final load of 5–6 (BcrMT1B), 8–9 (LlMT), 6–7 (SpMTA), 7 (XIMT), 3–4 (MacMT3), 4 (OsMTI-1B), 6 (Ec-1), 3 (PflQ2MT), 3–4 (SmtA), and 4 (TvMT) ions. This step corresponds to cluster(s) formation during which a much smaller number of new Cys residues are involved in Zn(II) coordination. The obtained data are highly valuable since they provide important insights into the Zn(II)-mediated folding of particular MTs and reveal similarities among all proteins, regardless of their origin. The results are also convergent with CD titrations of bacterial MTs, for which formation of the Zn_2_MT intermediate was observed in all cases, meaning that the first two ions are involved in coordination of a large amount of Cys residues, reflecting major structure change. Coordination of the next two Zn(II) ions results in the final cluster, easily observable by CD changes.^68^

### Probing the affinity range of Zn(II) sites in MTs

Although the literature is abundant in descriptions of metal binding properties of various MTs, it is, at the same time, poor in their thermodynamic characterization.^1,26^ Essentially it is due to the complexity of the coordination systems and lack or insufficient use of experimental methods with sufficiently high resolution to gain insight into such complicated systems as MTs. Basically, most of the methods are focused on determination of either average binding (*K*_b_^av^) or dissociation (*K*_d_^av^) constants determined by most of the methods such as spectroscopies, calorimetry or electrochemistry.^1,26,49,69,75^ An example of such an approach is UV-monitored spectroscopic pH titrations of MTs or their competition with chromogenic chelators. To shed more light on the problem of stability constants’ determination in this study we used several approaches, which allow the averaged and step dissociation constants of weak, moderate, and tight zinc sites to be investigated.

In all pH-dependent experiments, freshly prepared metal-free MTs were mixed with a slight molar excess of Zn(II) over the maximum Zn(II) saturation in 0.1 mM NaClO_4_ and pH was adjusted to 2.8–3. Such prepared solutions were titrated with freshly prepared 0.05–4 M KOH solutions in such a way that the pH increase was adjusted to absorption changes to obtain clear titration curves ending around pH 6.5 to avoid further protein oxidation. Supplementary Fig. S6 shows titrations of all Zn(II)-MTs over the applied conditions with fits to the logarithmic version of Hill equations.^43,76,77^ Data fitting allows one to obtain p*K*_a_ʹ values, which are averaged dissociation constants of thiol groups in MTs that compete with protons for metal ions. Basically, p*K*_a_ʹ values are shifted to lower values than p*K*_a_ values of free thiols, and the extent of this change is indicative of the affinity of the metal ion for protein (in fact for Cys-rich binding sites). At lower p*K*_a_ʹ values there are tighter metal bonds to MT. It must be underlined that in the case of MT, where Cys residues dominate, the pH-dependent spectroscopic effect corresponds to the formation of LMCT bands. The single transition at the pH titration curve indicates similar affinity of metal ions; however, in practice it shows only the binding of the tightest metal ions.^1^ Therefore any stability data based on pH titrations must correspond to the averaged value of tight binding sites. The binding of weaker Zn(II) ion(s) should be observable at slightly higher pH, but due to differentiated spectroscopic effects of LMCT for terminal and bridging M–S bonds (see earlier text) it is usually omitted or is visible as a continuous increase in absorption without noticeable inflection points as for tight binding sites where the formation of individual binding sites predominates.^1,28^ The continuous increase of absorbance, especially in higher pH, can also be a sign of protein precipitation, oxidation, deprotonation of media components, etc. Therefore additional approaches are usually required to say more about moderate or weak binding sites.^49^ In this study, all titration curves (Supplementary Fig. S6) show a single pitch and a slight continuous increase of absorbance. The order of obtained p*K*_a_ʹ values of investigated proteins from the tightest to weakest regarding Zn(II) affinity is as follows: TvMT < SmtA < Ec-1 < XIMT < PflQ2MT < LlMT < SpMTA < BcrMT1B∼MacMT3 < OsMTI-1B (Table 3). The values for Ec-1 and MacMT3 are highly similar to those obtained in the previous studies.^78^ To obtain any numeric values for *K*_d_ values of Zn(II)-MTs, knowledge of p*K*_a_ values of free thiols (p*K*_a_ of the spontaneous R-SH dissociation constant) would be needed, which is beyond the scope of this study.^1,3,4^ However, the relatively wide range of p*K*_a_ʹ values obtained here (0.85 log unit) indicates the significant difference in *K*_d_ values between investigated Zn(II)-MTs. Comparing this p*K*_a_ʹ range shift to other analogous values of Cys-rich zinc sites studied in the past, we may say that it would correspond to around ∼3.5 orders of magnitude difference in the p*K*_d_ value.^79^ However, it should be remembered that each binding site is different and different stabilization effects occur. Therefore, comparison of p*K*_a_ʹ values from MTs of different origin should be undertaken with a caution. Still, the performed experiments provide the important information that the p*K*_d_ value of zinc sites in bacterial MTs should be ∼3 orders of magnitude higher than corresponding values of animal and plant MTs (see following text). Notably, this conclusion is valid only for tight binding sites in MTs, since pH-dependent spectroscopic effects reflect mainly metal binding to those sites (see earlier).

**Table 3.** Stability constants of Zn(II)-MT complexes determined in this study using several approaches (details described in the text)^e^

		PAR		ZnAF-2F			
Metallothionein	UV titration p*K*_a_ʹ	−log*K*_d_^av^	−log*K*_d1_, −log*K*_d12_^av^ or −log*K*_d13_^av^	−log*K*_d_^weak^	−log*K*_d_^moderate^	−log*K*_d_^tight^	metal buffers −log*K*_d_^av^
BcrMT1B	4.97 (1)	11.47 (4)	**1**: 9.11 (1)	**1**: 8.03 (2)^a^	**2**: 9.80 (4)^a^	**4–6**: 11.5 (3)^b^	–
					**3**: 10.5 (2)^b^		
LlMT	4.83 (1)	10.69 (4)	**1–3**: 10.01 (4)	**1–3**: 6.75 (4)^a^	**4:** 10.0 (1)^b^	**6–9**: 11.5 (2)^b^	–
					**5**: 10.9 (1)^b^		
SpMTA	4.94 (2)	11.33 (1)	**1–2**: 10.56 (1)	**1**: 8.54 (3)^a^	**2**: 9.31 (4)^a^	**4**: 11.2 (2)^b^	–
					**3**: 10.5 (1)^b^	**5–7**: 11.5 (3)^b^	
XlMT	4.73 (3)	11.29 (3)	**1–2**: 10.52 (3)	**1**: 7.74 (6)^a^	**2**: 9.37 (9)^a^	**4–7**: 11.2 (4)^b^	–
					**3**: 10.1 (2)^b^		
MacMT3	4.97 (3)	11.03 (2)	**1–2**:10.73(2)	**1**: 8.81 (1)^a^	**2**: 9.9 (2)^b^	**3**: 11.4 (4)^b^	–
OsMTI-1B	5.04 (3)	10.78 (1)	**1–2**: 9.73 (1)	**1–2**: 7.76 (2)^a^	**3**: 10.1 (1)^b^	**4**: 11.7 (3)^b^	–
Ec-1	4.44 (2)	11.52 (2)	**1**: 9.67 (2)	**1**: 7.96 (6)^a^	**2**: 9.58 (9)^a^ **3**: 10.8 (1)^b^	**4–6**: 11.1 (2)^b^	–
PflQ2MT	4.75 (3)	13.40 (1)	**1**: 12.90 (1)	10.3 (2)^a^	*n.d.*	^c^	*n.d.*
SmtA	4.29 (3)	13.98 (1)	**1**: 13.36 (1)	11.2 (1)^a^	12.9^d^	^c^	14.68 (5)
TvMT	4.19 (2)	14.10 (1)	**1**: 13.49 (1)	10.9 (2)^a^	12.6^d^	^c^	14.3 (1)

^a^Values obtained from Zn(II) transfer to ZnAF-2F from fully Zn(II)-loaded MTs.

^b^Values obtained from Zn(II) titration to apo-MT with ZnAF-2F.

^c^Values were ignored due to underestimated affinity range.

^d^Value calculated (estimated) as the average of weak (MT competition with ZnAF-2F) and tight (metal buffer) dissociation constants.

^e^p*K*_a_ʹ denotes the average p*K*_a_ of thiols that are in competition with Zn(II). −log*K*_d_^av^, −log*K*_d1_, −log*K*_d12_^av^, and −log*K*_d13_^av^ correspond to the average dissociation constant of all zinc sites, the first step dissociation constant, the average value of −log*K*_d1_ and −log*K*_d2_, and the average value of −log*K*_d1_ to −log*K*_d3_, respectively. The values −log*K*_d_^tight^, −log*K*_d_^moderate^, and −log*K*_d_^weak^ correspond to dissociation constants of particular zinc sites (*K*_d1-_*_x_* where *x* is maximum Zn(II) load) grouped into tight, moderate and weak sites taken from two types experiments with ZnAF-2F (Zn(II) transfer from fully loaded MTs and Zn(II) competition for ZnAF-2F and metal free MT. Their numbering varies depending on MT and is presented in bold. −log*K*_d_^av^of metal buffers is an average constant of the Zn(II)-dependent events affecting CD signals. Numbers in parentheses correspond to the error of the last digit. *n.d.* denotes that the value was not determined due to technical problems or limitations.

**Fig. 5:**
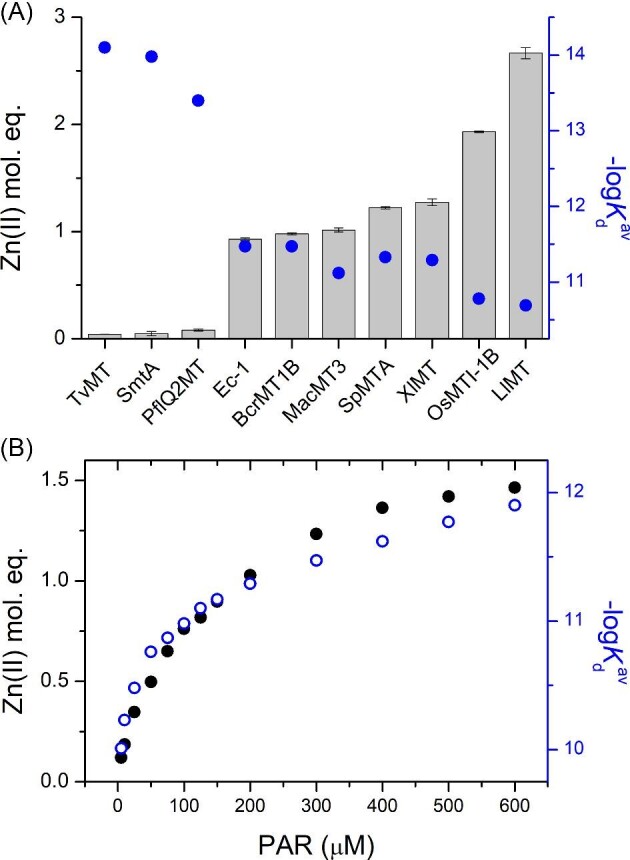
Competition of Zn(II)-loaded MTs with PAR. (A) Comparison of transferred Zn(II) mol. equivalents from 1 µM MTs to 200 µM PAR. The transfer was measured as absorbance increase at 492 nm and converted to ZnH_n_(PAR)_2_ complex concentration. Blue circles demonstrate −log*K*_d_^av^ values calculated based on the competition. (B) Transfer of Zn(II) from 1 µM XlMT to increasing PAR concentration (black circles) and calculated −log*K*_d_^av^ values based on these transfers (blue empty circles). All measurements were performed in 50 mM Na^+^-HEPES buffer, 100 mM NaCl, 100 µM TCEP, pH 7.4. Data are shown as means of *n* = 2 independent experiments + SD.

In contrast to pH titrations of Zn(II)-MTs, their competition with weak or moderate affinity chelators gives insights into the affinity of the weakest zinc sites in MTs. If the competitor is a chromophoric chelator, as in the case of PAR, this process is simple, and the whole method is very sensitive. The competition of Zn(II)-loaded MTs with PAR was performed in the presence of TCEP as a disulfide reducing agent of weak metal binding capacity.^80^ The Zn(II) transfer from MTs to PAR was monitored over time to reach a constant absorbance value. For animal and plant MTs this transfer was monitored for 10–30 min while for bacterial proteins this time was set as a minimum of 60 min. Absorbance values at 492 nm obtained after equilibration were converted to Zn(II) molar equivalents transferred during this process using the molar coefficient of ZnH*_x_*(PAR)_2_ complex 71 500 M^−1^⋅cm^−1^.^58^ The comparison of Zn(II) mol. eq. transferred to 200 µM PAR is presented in Fig. 5A, which indicates a significant difference between particular proteins. For instance, bacterial proteins were able to transfer from ∼25 to ∼75 times less Zn(II) than others, but among them the most tightly bound protein is TvMT (0.04 Zn(II) mol. eq.), then SmtA (0.05 Zn(II) mol. eq.) and PfQ2MT (0.08 Zn(II) mol. eq.). From animal MTs the lowest Zn(II) transfer was observed for BcrMT1B (0.98 Zn(II) mol. eq.), then SpMTA (1.22 Zn(II) mol. eq.), and XlMT (1.27 Zn(II) mol. eq.), while the highest was observed for LlMT (2.66 Zn(II) mol. eq.). Plant proteins show very similar profiles to animal proteins, indicating comparable affinities. The lowest amount of Zn(II) transfer was observed for Ec-1 (0.93 Zn(II) mol. eq.), then MacMT3 (1.02 Zn(II) mol. eq.), and the highest for OsMTI-1B (1.93 Zn(II) mol. eq.). As pointed out earlier, the absorbances of the ZnH*_x_*(PAR)_2_ complex at equilibria state and its known molar absorption coefficient and dissociation constant, allow the weakest dissociation constants of MT to be calculated. There are several ways to process such competitions, which rely on different dissociation models.^49,58,69^ Historically, however, the most inaccurate is the calculation of an average *K*_d_^av^ value of all zinc sites in MT.^1^ According to that, M*_x_*T molecules (*x* is the number of metal ions bound to thionein: metal free MT) is treated as a substrate and thionein as a product of one cooperative dissociation event. Based on the known Zn(II) mol. eq. transferred, *K*_d_^av^ is calculated.^49,69^ It should be mentioned that although this model was commonly used due to convenience, it is not fully correct, since in MTs + PAR equilibria metal-free protein is not formed due to insufficiently high affinity of PAR for extraction of Zn(II) from tight binding sites. Instead, metal-depleted species are formed due to stepwise dissociation processes. However, the calculated −log*K*_d_^av^ values presented in Table 3 indicate significant differences in affinities between bacterial and animal or plant MTs by 2.5–3.5 orders of magnitude. The presence of zinc sites with different affinities in one MT molecule would result in shifting the *K*_d_^av^ value as a function of competitor concentrations, since various sites from the weakest to moderate would be mobilized. We chose XlMT as an example and studied Zn(II) transfer to PAR from 5 to 600 µM followed by the determination of *K*_d_^av^ (Fig. 5B). The calculated −log*K*_d_^av^ values vary from 10.0 to 11.9 for applied conditions. The major shift in *K*_d_^av^ indicates clearly that various affinity sites are present in XlMT and that *K*_d_^av^ is not the best parameter for comparison of various proteins.^49^

Another dissociation model, which really reflects the situation occurring when investigating proteins during competition with PAR, is the stepwise model as described recently.^69^ According to that, for MTs which transfer up to 1 Zn(II) mol. eq. (BcrMT1B, Ec-1, PflQ2MT, SmtA, and TvMT) the *K*_d1_ constant was calculated. For proteins with Zn(II) transfer from 1 up to 2 Zn(II) mol. eq. (SpMTA, XlMT, MacMT3, OsMTI-1B) *K*_d12_^av^ values were calculated, which are average values of *K*_d1_ and *K*_d2_ only (these two events occur during PAR competition). For the weakest proteins, which demonstrate the transfer of 2 to 3 Zn(II) mol. eq. (OsMTI-1B and LlMT) *K*_d13_^av^ values were calculated, which are average values of *K*_d1_, *K*_d2_, and *K*_d3_. Calculated *K*_d1_, *K*_d12_^av^, and *K*_d13_^av^ values for all MTs are presented in Table 3 and exemplary calculations are presented in supplementary data. The obtained data show that bacterial MTs bind the weakest Zn(II) ions with femtomolar affinities, while plant and animal MTs bind them with subnanomolar affinities. Among plant and animal ones the tightest site(s) was found for SpMTA and XlMT, while the weakest were found for LlMT and BcrMT1B. It should be pointed out again that stability constants determined by MT competition with PAR represent the affinity of the weakest zinc sites, while pH titrations give insights into the affinity of the tightest sites (see earlier). Their correlation is not obvious since some MTs may contain very weak and tight sites, others not. Therefore, applying various approaches for determining zinc site affinities is essential.

**Fig. 6:**
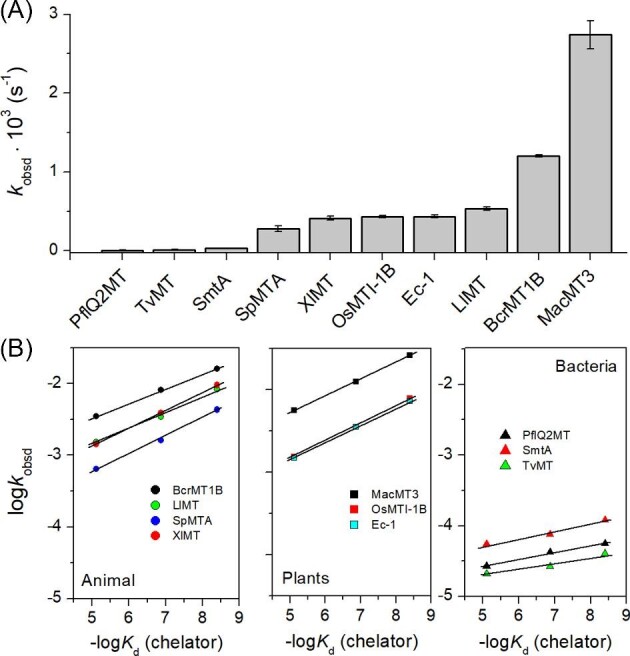
Rates of MT oxidation with DTNB revealing differences in Zn(II)-to-protein affinity and various Cys reactivity. (A) comparison of rates depending on the particular zinc MT. To determine *k*_obsd_ 1 µM MT was mixed with 1 µM DTNB in 50 mM Na^+^-HEPES, pH 7.4 (0.1 M NaCl) and the increasing concentration of TNB^−^ was monitored over time. (B) effects of weak and medium strong chelator (ATP, triphosphate, and NTA) on the rate of MT oxidation with DTNB. The *k*_obsd_ values were determined for the same samples as earlier but in the presence of 1 mM chelator. The affinity of each chelator was transformed to an apparent dissociation constant −log*K*_d_ (chelator) and plotted against log*k*_obsd_. Data are shown as means of *n* = 2 independent experiments + SD.

Another affinity test that has been applied here to probe zinc site affinities is oxidation of MTs by DTNB, similarly to the previously described approach, but in the presence of weak and medium strong chelating molecules such as ATP, triphosphate, or nitrilotriacetic acid (NTA). All these molecules are known to bind Zn(II) with 1:1 stoichiometry, and their apparent dissociation constant (−log*K*_d_) values at pH 7.4 are 5.1, 6.9, and 8.4, respectively.^81,82^ Rates of MT oxidation by DTNB in the presence of chelating molecules are higher due to their competition with the weakest sites. Mobilized Zn(II) either reveals unbound Cys residue(s), which are prone to faster oxidation or changes protein structure in such a way that oxidation occurs more rapidly due to higher dynamics of protein. Figure 6A compares the first *k*_obsd_ values of MTs’ oxidation without chelating molecules. It shows that the most resistant to oxidation are bacterial MTs due to their stability and compact structure containing a hydrophobic core. Plant and animal MTs are oxidized faster, but the rate of this reaction varies significantly from the slowest SpMTA to the fastest MacMT3. Note that the oxidation rate does not necessarily reflect affinity of MTs, but rather their natural tendencies for oxidation. However, the oxidation performed in a set of chelating agents reveals information about the range of affinities. Figure 6B presents relations between log*k*_obsd_ values and −log*K*_d_ of chelators in whose presence oxidation reactions were performed. Interestingly, for all MTs this relation is linear. Oxidation of animal and plant MTs is highly similar, which is represented by the same range of log*k*_obsd_ values and comparable slope of obtained linear relations. It indicates that MTs from these organisms have similar affinities and reactivity (tendency for oxidation). These conclusions match those for mammalian MTs studied previously.^48^ Bacterial MTs are certainly different. The linear relations are shifted by two to three orders of magnitude compared to animal and plant proteins. Moreover, their slope is clearly lower. Both these observations are indicative of much higher thermodynamic stability of bacterial proteins and lower reactivity. However, the fact that oxidation rates change from ATP to NTA indicates that zinc sites of these proteins compete somehow with moderate zinc chelators, indicating that at least some binding sites might be weaker from others.

### Insights into weak, moderate, and tight binding sites of MTs

**Fig. 7:**
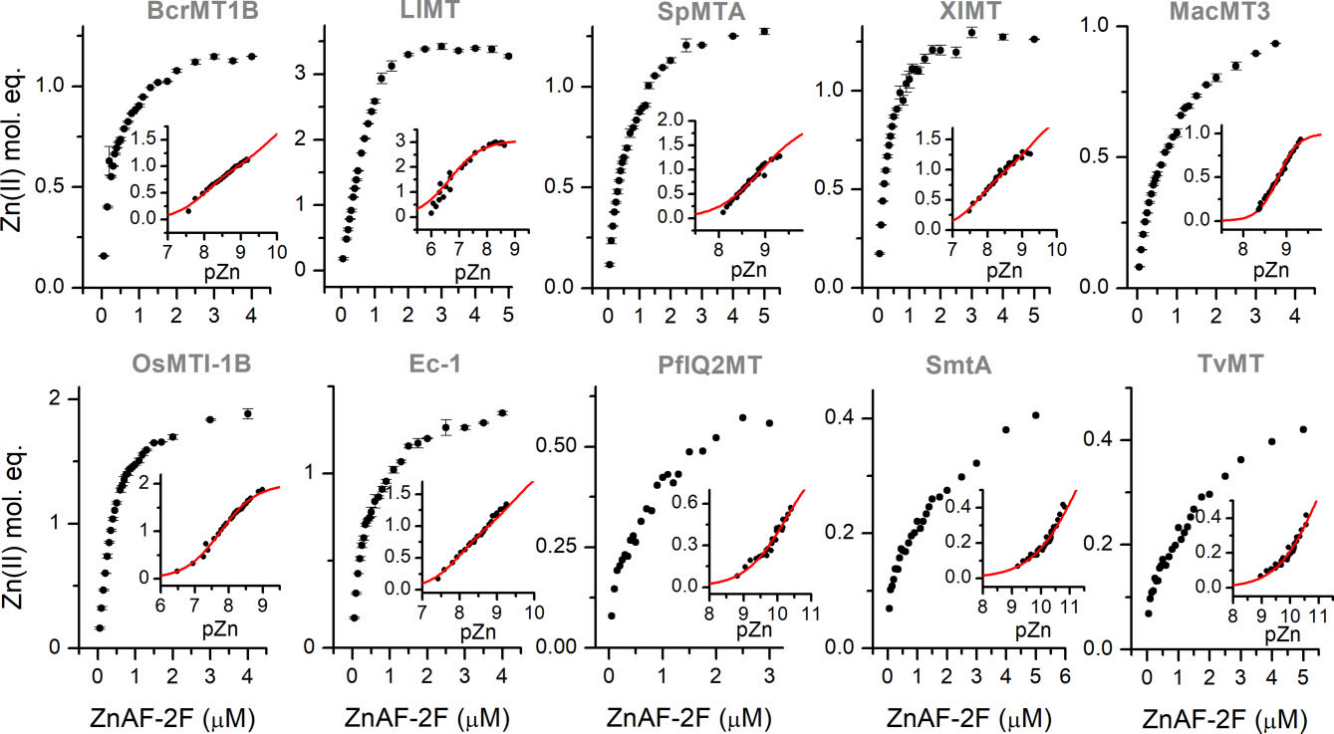
Zn(II) transfer from MTs to ZnAF-2F. MT (0.5 µM) was mixed with 0–5 µM ZnAF-2F in 50 mM Na^+^-HEPES (100 mM NaCl, 500 µM TCEP), pH 7.4, and incubated for 90 min. Measured fluorescence was calibrated with ZnSO_4_ or EDTA and presented as transferred Zn(II) eq. Insets represent −log[Zn(II)]_free_ (pZn) values as a function of transferred Zn(II) mol eq. fitted to one- or two-site model. Data are shown as means of *n* = 2 independent experiments + SD.

The results described in the previous section indicate that animal and plant MTs contain weak and tight affinity sites, while bacterial MTs bind Zn(II) tighter. Even for them, affinities of zinc binding sites may be differentiated. To shed much more light on these observations and gain deeper understanding of the thermodynamics of MTs, especially animal and plant, we applied here a highly sensitive fluorescent probe, ZnAF-2F. It forms a 1:1 complex with Zn(II), whose apparent *K*_d_ at pH 7.4 is 5.5 × 10^−9^ M.^83^ Its signal increase upon Zn(II) coordination allows for the determination of free Zn(II) even at a low pM range. Due to that sensitivity, we applied ZnAF-2F for two different approaches to obtain quantitative data on weaker, moderate and high-affinity zinc sites. In the first approach 0.5 µM MTs were incubated with increasing concentrations of ZnAF-2F from 0.05 to 5 µM (or 4 in the case of lower Zn(II) content in MT) for 90 min and fluorescence was measured. This time was sufficient to reach an equilibrium between reagents. Increased fluorescence at 516 nm was converted to transferred Zn(II) mol. eq. by its comparison to the value obtained by NPSC (2-nitrophenylselenocyanate) oxidation of all thiols (Fig. 7). This reagent causes dissociation of all Zn(II) ions in the applied conditions.^84^ The results show that the transfer is observed for all MTs including bacterial ones. However, in that group the transfer was significantly low, indicating 0.4–0.5 Zn(II) mol. eq. mobilization. The rest of the MTs transfer either 1 (MacMT3) or more than 1 Zn(II) mol. eq. The largest amount of transferred Zn(II) was observed for LlMT, which demonstrated mobilization of almost 3 Zn(II) mol. eq. In order to obtain stability data, fluorescence intensities after incubation were converted to free Zn(II) concentrations by additional calibration with ZnSO_4_ and EDTA (see section “Experimental procedures”). Insets of Fig. 7 demonstrate the functions of −log[Zn(II)]_free_ (pZn) and Zn(II) transferred. These data show only a fragment of the larger picture, in which −log*K*_d_ of a particular zinc site might be determined for each site.^49^ Here, having only a fragment of that picture (which constitutes a limitation of the method and approach) we fitted the obtained data to a one- (*K*_d1_ only) or two-site (*K*_d1_ and *K*_d2_) binding model, allowing determination of −log*K*_d_ values of the weakest and the second weakest binding site, as presented in Table 3. In the case of LlMT one binding site model was applied due to cooperative transfer (Fig. 7). These values indicate that the weakest zinc site is in the nanomolar range for all animal and plant MTs. These results are convergent with data obtained previously for human MT2, which binds seven Zn(II) ions in three groups of affinities from pico- to nanomolar range.^48^ The second dissociation constant, *K*_d2_ (if applicable), was correspondingly higher (Table 3). Interestingly, the correlation of −log*K*_d1_, −log*K*_d12_^av^, or −log*K*_d13_^av^ from PAR experiments with −log*K*_d_ values of the weakest sites from ZnAF-2F competition indicates a high linear correlation (Supplementary Fig. S7, *R*^2^ = 0.91). It is because in both measurements, the weakest sites are mobilized. Only LlMT and MacMT3 deviated from the trend line, although still within the 95% confidence interval. It may result from specific interaction of certain MTs with PAR or ZnAF-2F; however, the second was present in ∼100-fold lower concentration. Another reason is the fact that dissociation constants for both chelators are defined slightly differently.

**Fig. 8:**
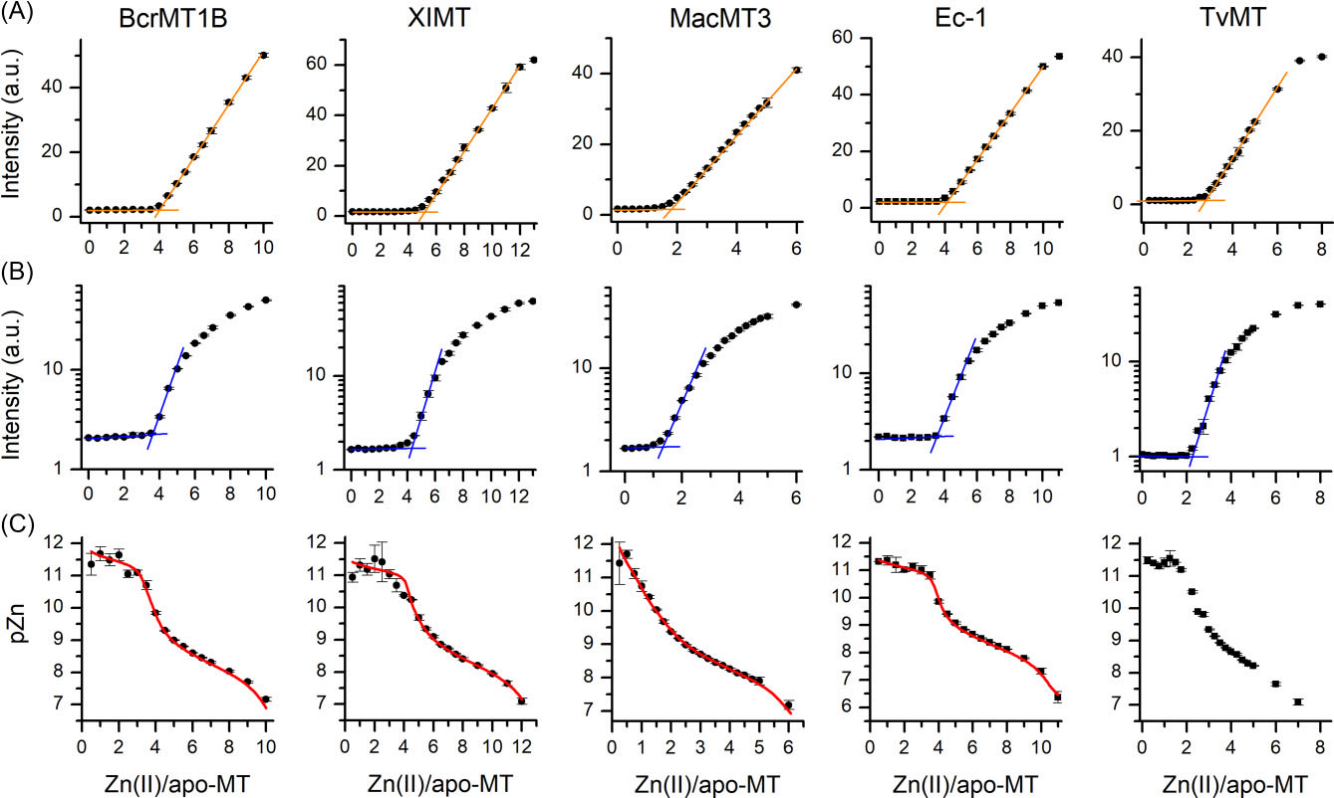
Titration of metal-free BcrMT1B, XlMT, MacMT3, Ec-1, and TvMT with ZnSO_4_ in the presence of ZnAF-2F. (A) 0.5 µM apo-MT and 2–3.5 µM ZnAF-2F (concentration varied depending on protein) in 50 mM Na^+^-HEPES, 100 mM NaCl, 500 µM TCEP. Orange line helps demonstrate titration end points. (B) Semilog plots of the same data are presented to visualize changes during titration. Blue lines help to determine the number of tight zinc sites. (C) Fluorescence responses were calibrated to obtain [Zn(II)]_free_ concentrations and are shown as a pZn function of Zn(II)/apo-MT. Red lines demonstrate simulations based on calculated constants. −log*K*_d_ values obtained from data fitting are presented in Table 3 (see section “Experimental procedures”).^47^ Data are shown as means of *n* = 2 independent experiments + SD.

In the second ZnAF-2F-based approach, we used apo-MTs instead of fully Zn(II)-loaded MTs. For that purpose 0.5 µM MT was incubated with 2–4.5 µM ZnAF-2F and titrated with ZnSO_4_ up to 8–13 Zn(II)/apo-MT ratios in such a way that each sample was prepared in a separate cuvette. All samples were incubated in the dark for 90 min and measured. Figure 8A and Supplementary Fig. S8A demonstrate ZnAF-2F fluorescence profiles depending on the increasing Zn(II)-to-protein molar ratios. All these profiles indicate practically unchanged intensities, which during titration drop significantly at a certain Zn(II)/apo-MT. It indicates almost no competition at the first stage of the titration due to efficient Zn(II) binding to tight binding sites in MTs. However, only semilog plots of these fluorescence intensities (Fig. 8B, Supplementary Fig. S8B) shed more light on the competition process. For instance, in the case of BcrMT1B four Zn(II) ions are bound very tightly, observable by only a minor fluorescence increase. Above a Zn(II)/apo-MT ratio of four, there is visible a linear intensity increase, which becomes round at a certain Zn(II) content. The linear region indicates clear competition between moderate binding sites with ZnAF-2F, while the rounded part corresponds mostly to titration of the free probe and partially weaker binding sites. It should be noted that under the applied conditions it is impossible to determine affinities of the weakest site(s), which was determined in the experiment where Zn(II) transfer from Zn(II)-loaded MT was analysed (see earlier). Briefly, semilog plots besides BcrMT1B show the presence of 4 (LlMT), 4 (SpMTA), 4 (XlMT), 1 (MacMT3), 1 (OsMTI-1B), 4 (Ec-1), 2 (PflQ2MT), 2 (SmtA), and 2 (TvMT) tight binding sites. Unequivocal determination of the number of moderate binding sites is difficult due to the lack of clear titration endpoints, yet still possible. Importantly, the number of tight binding sites in MTs corresponds very well to the data from DTNB-monitored titrations (see discussion in the following text). Calibration of ZnAF-2F intensities, similarly to the first approach, allowed us to plot them as Zn(II)/apo-MT functions of pZn as presented in Fig. 8C and Supplementary Fig. S8C. To determine *K*_d_ values of tight and moderate binding sites, these plots were processed using HySS software in such a way that all known stability constants and concentrations of ZnAF-2F and MTs were fixed and only *K*_d_ values of tight and moderate sites were refined in manual mode to match experimental points, similarly as in previous human MT2 studies.^48,85^ Based on these fixed (ZnAF-2F) and determined *K*_d_ (MT) values the best model was plotted in Fig. 8C and Supplementary Fig. S8C as a red line. Note that under used here conditions not all Zn*_x_*MT are formed [weakest one(s)] and their constants were not calculated in this approach. Calculated dissociation constants of tight and moderate sites are grouped regarding their affinity and presented in Table 3. They show that all animal and plant MTs demonstrate low picomolar binding sites (log*K*_d_^tight^ from 11 to 11.6) similarly to mammalian MTs.^48^ The same values were obtained for bacterial proteins, but were ignored here (no data in Table 3 and fits in Fig. 8C and Supplementary Fig. S8C) since they are underestimated (in terms of −log*K*_d_ values) due to insufficient sensitivity of ZnAF-2F under the concentrations used. These values were instead determined by the competition with tighter chelators (see following text). The −log*K*_d_ of moderate sites of animal and plant MTs varies from 9.3 to 10.95, showing indeed moderate character.

### High-affinity zinc sites of bacterial MTs

**Fig. 9:**
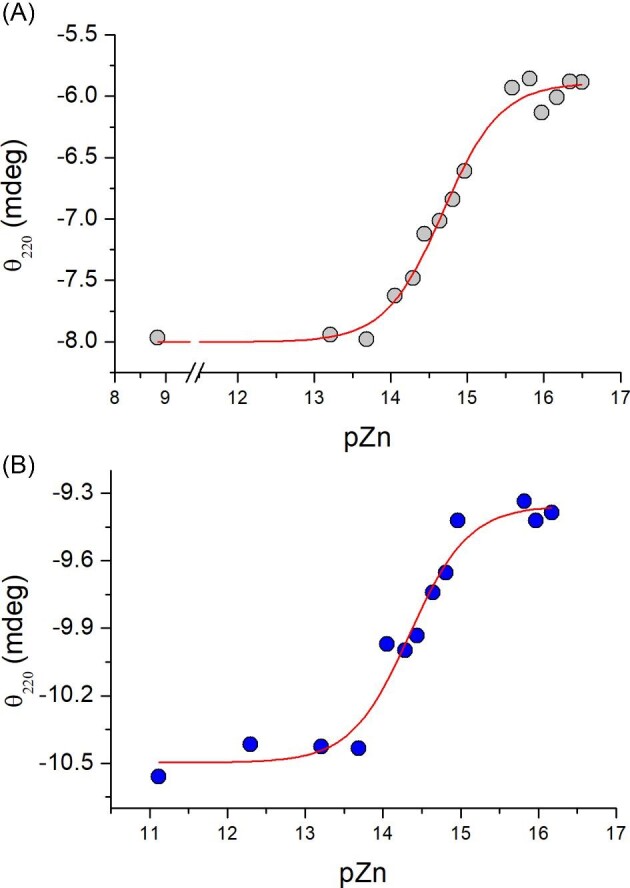
Isotherms of Zn(II) binding to (A) SmtA and (B) TvMT proteins in a set of metal buffers in 10 mM Tris-HCl, 100 mM NaClO_4_, 360 or 400 µM TCEP, respectively, pH 7.4. Data were fitted to Hill's equation.

All obtained data indicated that bacterial MTs are very different from animal and plant counterparts. They show that these proteins are highly stable in terms of thermodynamics and reactivity. The high stability is related to well-folded structures (determined by NMR or predicted), containing GATA-type zinc finger fold.^67,68^ Its formation upon Zn(II) complexation results in the numerous intramolecular stabilization interactions affecting overall free energy.^77^ The findings support our previous research on characterization of zinc finger domains, where their affinities were determined using a series of metal buffers composed of high-affinity complexones (common chelators).^43,76,79^ CD spectra presented in Fig. 3 show that SmtA and TvMT demonstrate enough high changes in ellipticity that could be used in CD-monitored competition studies with complexones. In contrast, preliminary studies have shown that data obtained for PflQ2MT show changes that are in the range of experimental error and were not taken into consideration. Therefore 20 µM apo-SmtA and TvMT were incubated with zinc buffers composed of *N*-hydroxyethyl-ethylenediamine-triacetic acid (HEDTA), EDTA, and *N,N,N’,N’*-Tetrakis(2-pyridylmethyl)ethylenediamine (TPEN) partially saturated with ZnSO_4_ (see section “Experimental procedures”). These chelators are able to buffer free Zn(II) in the range from nano- to attomolar, since their apparent *K*_d_ values at pH 7.4 are 6.6 × 10^−13^, 2.3 × 10^−14^, and 6.4 × 10^−16^ M, respectively.^81,82^ Samples were incubated for 8 h, which was sufficient time for the equilibration. Figure 9A and B show ellipticity changes at 220 nm for SmtA and TvMT, respectively, as a function of −log[Zn(II)]_free_. Data fitting to Hill's equation demonstrated −log*K*_d_ almost in the same range, being 14.7 and 14.3, respectively. Due to insufficient resolution of the applied approach it is difficult to say whether the obtained constants are averages of all sites or more preferentially represent those site(s) that cause the largest conformational change recorded by the CD signal (tighter ones). Previous NMR study has shown that zinc finger fold in SmtA is formed when site A is occupied. Demetalation studies have shown that this is the last site to leave the molecule when SmtA is reacted with EDTA.^86^ It suggests that *K*_d_ values obtained spectrobolometrically describe the affinity of the most tight site(s) in the zinc finger fold of SmtA and TvMT. Bacterial MTs bind Zn(II) significantly more tightly than the most stable zinc sites of animal and plant MTs. This range of affinity (*K*_d_ value) is similar to free Zn(II) concentration ranges for zinc sensor protein SynZur binding to *PznuA* and *PbmtA* promoters very recently reported in a marine cyanobacterium.^87^ It indicates potential importance of MTs in the regulation of Zn(II) content in bacteria (see discussion in the following text).

### Relations between Zn(II)-mediated folding and zinc site affinities

The results obtained in this study shed new light on the whole zinc-binding family of MTs. They indicate that not only human or mammalian MTs are capable of Zn(II) binding with various nano- to picomolar affinities. As shown in several examples, MTs from animals (nonvertebrate) and plants despite obvious differences display very similar Zn(II) binding affinities as mammalian ones. It demonstrates that mechanisms behind zinc site stability differentiation are either the same or very similar. Several decades of investigations on mammalian MTs have shown that the binding of Zn(II) differs from Cd(II) in terms of metalation pathways (not final structures).^1^ As recently demonstrated, Zn(II) ions bind first in human MT2 to two domains independently and then complement initially formed cores, in contrast to Cd(II), which first form the α- then β-cluster.^28^ This way of folding results in differentiated Zn(II) affinity, which derives from intra- and interdomain interactions in MT2. As proven recently, the connection of both α- and β-domains has a serious impact on lowering the affinity of some Zn(II) sites and impacts domain folding and in consequence metal-to-particular site affinity.^69^ In this study various MTs, containing one to three domains, were chosen and probed for their affinity to Zn(II). The collected data, especially those for ZnAF-2F, show that in all of them affinities of zinc sites are differentiated, although to varying degrees, indicating enhanced zinc buffering capacity.^1^

Starting with animal MTs, BcrMT1B binds six Zn(II) ions with a wide affinity range. DTNB oxidation-monitored Zn(II) titration has shown that binding the first four Zn(II) ions significantly decreased the oxidation rate of thiols, meaning that these ions binding to MT form independent or modestly clustered sites (Fig. 4). Similarly, ZnAF-2F competition has shown tight (low picomolar) binding of 4 Zn(II) mol. eq. (Fig. 8). Based on the structure of BcrMT1B and analogy to two-domain human MT2, we may suggest that two pairs of Zn(II) ions bind to the α- and β-domain, respectively, and cover the maximal number of Cys residues.^48^ The remaining two Zn(II) ions occupy both domains forming the α- and β-cluster. It significantly lowers their affinity (Table 3). LlMT is very different from all other proteins studied here, since it forms three α1, α2, and β domains containing three metal ions.^20,88^ The binding of the first three to four Zn(II) ions causes a significant decrease of thiol oxidation by DTNB (Fig. 4), but a visible lack of reactivity occurs at 9 Zn(II) eq. The competition of apo-MT with ZnAF-2F shows that ∼6 ions bind efficiently, of which ∼4 ions bind very tightly. On the hand, Zn(II) transfer from Zn_9_LlMT to ZnAF-2F indicated spontaneous mobilization of ∼3 Zn(II) ions (Fig. 7). This altogether shows the complicated system in which the first groups of Zn(II) ions bind with picomolar affinity, then with moderate affinity, −log*K*_d_^moderate^ = 10.95, continue filling the domains, while the last three ions, with relatively weak affinity of −log*K*_d_^weak^ = 6.75, close the three clusters of LlMT (Table 3). SpMTA and XlMT bind seven Zn(II) ions in two M_4_Cys_11_ and M_3_Cys_9_ domains similarly to the mammalian one as shown by structural investigations, but SpMTA contains domains placed oppositely to XlMT and mammalian ones.^62^ All of them bind tightly four Zn(II) ions as shown by DTNB oxidation and apo-MTs’ competition with ZnAFs (Fig. 4, 7, and 8, Supplementary Fig. S8). Similarly to human MT2, two Zn(II) ions bind with moderate affinity (Table 3). The last one binds with nanomolar affinity, being in a similar range as the human ones, although that for SpMTA is the tightest from among all animals studied (Fig. 7; Table 3).^49^ It may come from swapped domains and a different intramolecular interaction pattern.^69^

From studied plant MTs only Ec-1 shows some structural similarities to animal ones, since it binds six Zn(II) ions in two γ- and β_E_-domains, but this binding is significantly different. The N-terminal γ-domain forms the Zn_2_Cys_6_ cluster, while β_E_ forms two independent cores with one ZF-like ZnCys_2_His_2_ and the Zn_3_Cys_9_ cluster common in MT.^63,89,90^ Our study confirmed the binding of the same number of Zn(II) ions and showed that the first three are bound with high affinity. The fifth (moderate) binds with −log*K*^moderate^ = 9.58, while the sixth (weakest) binds with −log*K*^weak^ = 7.96 (Fig. 8; Table 3). Based on structural and thermodynamic features of this MT, it is difficult to say how Zn(II)-mediated folding may occur. More likely the first three Zn(II) ions bind to three centers in both domains in such a way that two of them completed the last segment similarly to the β-domain of mammalian ones. Then, the remaining three Zn(II) ions complete segment, forming clustered cores. The next plant MT, MacMT3, binding three or four Zn(II) ions (at metal excess), forms in the first case one independent ZnCys_4_ site at the N-terminus, while the remaining two ions are bound in the Zn_2_Cys_5_His_2_ cluster at the C-tail. In the case of the Zn_4_MacMT3 form, both the N- and the C-terminal Cys-rich regions are combined into a single cluster containing four Zn(II) ions.^63,64^ In our study, the Zn_3_MacMT3 form was formed during reconstitution, while the formation of Zn_4_MacMT3 was observed in spectroscopic titration. The fourth Zn(II) ion, Zn_4_MacMT3, more likely binds with even lower affinity and therefore is lost during SEC purification during reconstitution. Data obtained in this study for MacMT3 show that one Zn(II) ion binds tightly with low picomolar, one with high picomolar (moderate) and the last with nanomolar affinity, as shown by DTNB oxidation and ZnAF-2F competition (Fig. 4, 7, and 8; Table 3). The binding of Cd(II) to MacMT3 is different, showing efficient coordination of four Cd(II) ions in one Cd_4_Cys_9_His_2_ cluster.^63^ OsMTI-1B has been much less investigated with Zn(II) to date compared to the other MTs chosen here.^65,91,92^ It binds four Zn(II) ions in unknown structure, although its amino acid sequence similarity to MacMT3 may suggest the formation of two cores at both protein tails, such as Zn_2_Cys_6_ and Zn_2_Cys_6_His_2_ (Fig. 1).^65^ Since structural data are unavailable, we cannot speculate about clustering or formation of an independent site. What is new from our study is that two Zn(II) ions bind Zn(II) tightly with picomolar affinity, while the remaining two ions coordinate with nanomolar affinity (Fig. 4 and 7, Supplementary Fig. S8; Table 3). Lack of a moderate site may, however, suggest different metalation pathway compared to MacMT3.

Bacterial MTs are the most folded proteins of all those investigated here (Fig. 3, Supplementary Fig. S5), which form the most stable complexes with Zn(II) (Table 3). Careful investigations showed that such rigid proteins bind Zn(II) ions in a step-binding manner like animal and plant ones, but this binding is shifted to higher affinities. Both DTNB-promoted oxidation of partially metalated PflQ2MT, SmtA and TvMT and their CD titration with Zn(II) indicated that the first two Zn(II) ions cause a significant structural change of these proteins. The remaining two ions, but in the case of PflQ2MT only one, form a final cluster (Fig. 3 and 4).However, kinetic studies on fully loaded SmtA by external chelators indicated that one of four sites (site C) is removed first before the others, and this site is absent in PflQ2MT.^86^ It is worth mentioning that this conclusion was made based on kinetic intermediates, not thermodynamic equilibrium.

Competition with PAR (Fig. 5A) and ZnAF-2F (Fig. 8) indicated that all bacterial MTs bind Zn(II) very tightly. In the case of ZnAF-2F it was found that ∼0.5 Zn(II) mol. eq. is transferred under the applied conditions, but much less was transferred in the case of PAR competition. It means that proteins must contain a relatively moderate affinity zinc site that is able to compete with these zinc probes. Zn(II) titration of apo-MTs + ZnAF-2F confirms the presence of two tight sites, but the dissociation constant values obtained from that experiment are overestimated since that probe is not able to compete with the tightest zinc sites as shown by CD-monitored competition with TPEN and EDTA (Fig. 9; Table 3). Therefore the most appropriate affinity model assumes two low femtomolar zinc sites and one (weakest) in the low picomolar range. In the case of SmtA and TvMT there is one more zinc site, whose affinity is between extreme (weak and tight) ones, more likely in the high femtomolar range (Table 3). Regardless of methodological limitations it is clear that that bacterial MTs demonstrate quite similar mechanisms of zinc metalation as animal and plant counterparts with significantly shifted affinities towards higher values. This shift is due to more pronounced metal-coupled folding, which results in more stabilizing interactions formed during Zn(II) coordination, which impact overall Gibbs enthalpy of Zn(II) complexation.^77^

### Differentiated affinities determine MTs’ zinc buffering properties

**Fig. 10:**
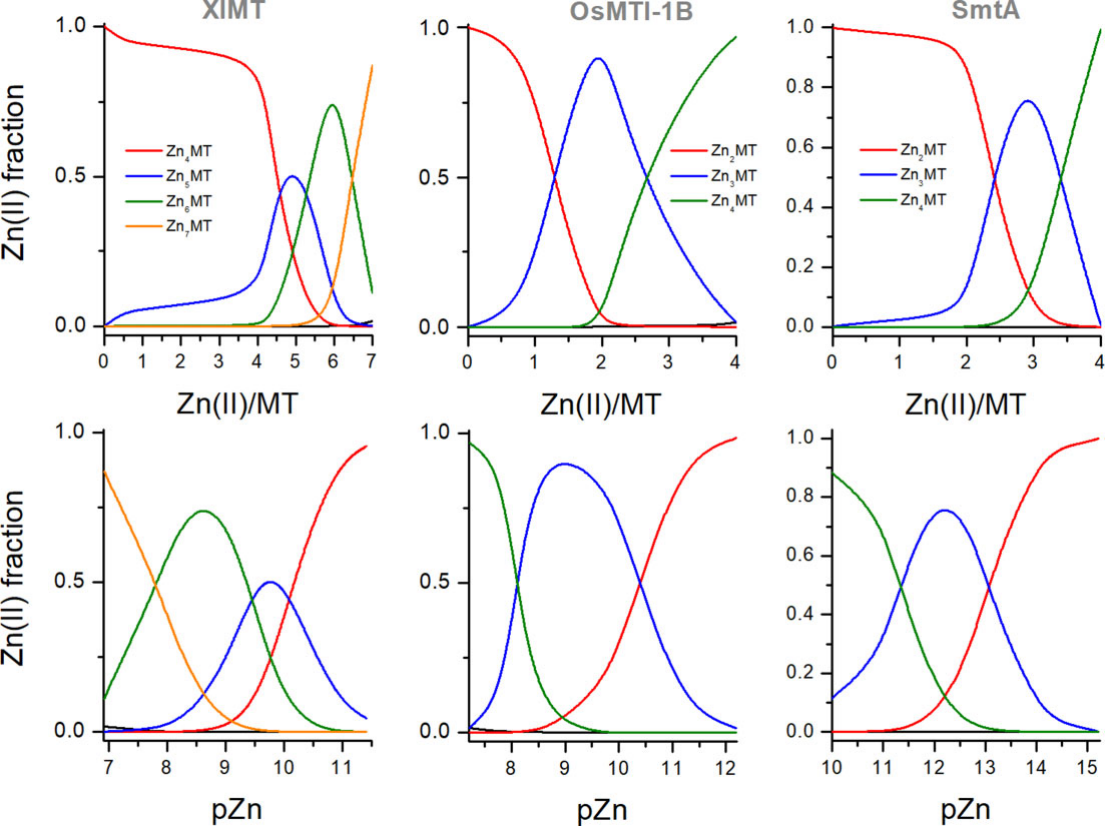
Zn(II)-MT species distributions of XlMT, OsMTI-1B and SmtA. Distributions (upper panel) were plotted using HySS software based on dissociation constants from Table 3 (for ZnAF-2F and metal buffers). Lower panel presents the same Zn(II) molar fractions as a function of pZn (−log[Zn(II)]_free_). Faintly visible black line shows free Zn(II) distribution.

Without a doubt, the most remarkable conclusion of this study is that the whole family of MTs, regardless of origin, demonstrates a stepwise Zn(II) metalation mechanism with significantly differentiated affinities. Taking into account that all these proteins bind several ions per molecule with various affinity, it is not surprising that all of them show high zinc buffering capacity, which allows free Zn(II) concentrations to be maintained within a certain physiologically relevant range. Based on stability constants presented in Table 3, it is now possible to plot zinc speciation, which very precisely shows distribution of molar fractions for all Zn*_x_*MT species identified for a particular protein. Figure 10 shows examples of such speciation for animal (XlMT), plant (OsMTI-1B), and bacterial (SmtA) MTs. Figure 10A indicates the presence of particular species at various Zn(II)/apo-MT ratios, while Fig. 10B shows the same fractions as a function of pZn. The latter one is especially interesting, since it clearly shows at which free Zn(II) concentrations particular MT species function. For example, in the case of XlMT, Zn_4_MT dominates up to a Zn(II)/apo-MT molar ratio of 4. Above that value consecutive Zn_5_MT, Zn_6_MT, and Zn_7_MT are present, whose relative molar fractions are regulated by Zn(II) content. Interestingly, species Zn_6_MT and Zn_5_MT are present with the highest content at pZn 8.5 and 9.6, respectively, indicating this region of free Zn(II) as the most efficiently buffered. It is difficult to correlate these values with free zinc concentration in *Xenopus laevis* due to the lack of systematic studies in this area. However, zinc proteins, including zinc finger factors (e.g. TFIIIA) and zinc transporters, bind Zn(II) similarly to mammalian proteins, indicating the same range of affinities.^93–95^ In the case of LlMT, the buffered pZn range is wider than for other animal MTs. It may be caused by the additional domain (compared to two-domain MTs) where binding of the last three Zn(II) ions occurs at a very low nanomolar range. This species has developed a mechanism against the damaging effects of natural stress factors such as anoxia and freezing. During arrest these factors and sudden reintroduction of oxygen cause ROS (reactive oxygen species) to appear but also overexpression of MT.^96^ MTs can counteract the harmful effects of ROS on cells by being ROS scavengers due to the large amount of cysteine residues, and the release of Zn(II) can affect the activation of enzymes related to protection against ROS, e.g. superoxide dismutase (SOD).^95,97^ LlMT is an unusual protein compared to others due to its three-domain structure. Evolutionarily, MTs are observed to undergo reshuffling, rearrangement, multiplication or formation of *de novo* domains, which provides new functional abilities, such as increasing the frequency of metal ion binding or increasing the affinity for a specific metal ion.^98^ In this way LlMT increases the buffering capacity and range of buffered pZn compared to two-domain MTs. Moreover, such an evolutionary procedure in the form of increasing the size of a given protein is more energetically advantageous than gene duplication, as was proven during research on yeast.^88,99^ Much more is known about zinc homeostasis in mammals, where MTs play a central role in zinc and copper metabolism.^1^ Their differentiated Zn(II) affinity allows them to accept an excess of metal ions or donate them when required. In all these processes the free Zn(II) concentration is kept within a narrow range of hundreds of pM. However, upon zinc signals or local fluctuations, its concentration rises, playing a signaling function.^40,44,100^ How the MT-related homeostasis occurs in animals other than mammals remains poorly understood.

The speciation of *Oryza sativa* MT (Fig. 10), despite its structural difference, indicates that zinc buffering occurs in a very similar range of free Zn(II) compared to animal MTs, only slightly shifted to higher values. The same is true for MacMT3 and Ec-1 proteins. It is difficult to say how zinc buffering properties of plant MTs are connected with their roles but definitively besides being involved in zinc metabolism or its accumulation, plant MTs are involved in protection processes occurring during tissue damage, as well as during biotic and abiotic stress.^101–105^ They are involved in the protection against pathogens or insects in various mechanisms in a direct way by locally increasing the concentration, as well as activation of Zn(II)-dependent proteins responsible for protection against pests, e.g. SOD, transcription factor activation, or carbonic anhydrase, which is responsible for defense using salicylic acid.^106,107^ Therefore, it seems that the functions of MTs are primarily related to the maintenance of zinc (and copper) homeostasis but, if necessary, play a role in toxic metal detoxification together with PCs.^108^ Definitely, coordination dynamics and various affinities of bound Zn(II) ions help plant MTs to be involved in many Zn(II)-related processes.

Speciation of Zn(II)-SmtA species presented in Fig. 10 is similar to other MT groups, but shifted to lower free Zn(II) concentrations. The Zn_2_SmtA species dominates at pZn 10, while Zn_3_MT dominates at 12.1, indicating that the zinc buffering range occurs between 9 and 14 on the pZn scale. The fact that bacteria bind Zn(II) more tightly is not new, but the literature is relatively poor regarding Zn(II) affinity studies of bacterial MTs.^68,73,109^^–^^112^ Total zinc concentration in bacterial cells varies from 10^−4^ to 10^−3^ M, while free Zn(II) concentration was found recently to vary from 10^−14^ to 10^−12^ M.^111,113^^–^^114^ This range very well matches the pZn range, where various Zn*_x_*SmtA species (but also Zn*_x_*PflQ2MT and Zn*_x_*TvMT) are present, suggesting that Zn(II)-depleted MT species might be involved in Zn(II) ion buffering and distribution.^115^ Oceans where marine cyanobacteria are present exhibit very low concentrations of zinc, varying from the pico- to nanomolar range.^116^ The concentration is even lower in some bodies of water such as the Sargasso Sea.^117^ Recent studies on *Synechococcus* sp. WH8102 demonstrated that the zinc sensor protein Zur activates MT gene expression, which increases cellular zinc accumulation by two orders of magnitude when zinc is available.^87,112^ Zinc in marine cyanobacteria is necessary for carbonic anhydrase involved in carbon accumulation and acquisition of phosphorous from organic substrates.^118^ Moreover, a study on PflQ2MT showed that its expression appears at the stationary growth phase and has an influence on long-term viability thanks to storage or sequestration of Zn(II) ions.^72^ This indicates a dynamic system, in which not only zinc accumulation but also its mobilization and transfer to zinc or zinc-dependent proteins are critical. It might be possible if Zn(II) is bound by MT with differentiated affinity, which matches the cellular free zinc concentration—exactly as was observed in this study.

## Conclusions

Binding of essential metals, such as zinc and copper, their handling and buffering of their concentrations are the primary functions of MTs from various organisms, without diminishing their role in the sequestration of toxic metal ions. The latter is especially important for those organisms that are exposed to toxic metal ions. The research presented here demonstrated that MTs from distinctly different organisms share similarities of Zn(II) binding, adding new insights into metabolism of this element. Animal (including mammals) and plant MTs bind Zn(II) with a similar range of affinities varying from the pico- to nanomolar range. It is especially important, since free Zn(II) concentrations and fluctuations found in eukaryotic cells occur exactly in this range of concentrations. Bacterial MTs demonstrate similar properties, but their affinity towards Zn(II) is shifted to higher values, allowing for its binding in the range from attomolar to low picomolar. Exactly this range of free Zn(II) was found in bacterial cells. Although our knowledge on Zn(II)-dependent processes is still fragmentary, it becomes clear that MTs due to their differentiated affinities and significant buffering properties are able to accept or donate Zn(II). It guarantees the availability of zinc for cellular processes protecting them against zinc toxicity and deficiency at the same time. As discussed, MTs’ zinc buffering properties are due to their folding mechanisms, which seems to be substantially different from Cd(II), e.g. indicating that buffering and sequestering properties might be performed within one type of MT molecule. Given the diversity of this group of proteins, the conclusions presented here apply to the analysed proteins and do not necessarily apply to every MT.

## Experimental procedures

### Materials

All reagents used in this study were of high purity and purchased from Acros Organic, Merck (Sigma-Aldrich), Roth, BioShop, Bio-Rad, VWR International, Witko, STANLAB, TCI, Lab Empire, Iris Biotech GmbH, New England BioLabs. All pH buffers prepared with Milli-Q water were incubated with Chelex 100 resin to eliminate trace metal ion contamination. More detailed information about the used materials is listed in the Supplementary data.

### Expression and purification of MTs

The cDNA encoding all investigated proteins was synthesized on request by GenScript (USA) and cloned into the pTYB21 vector using SapI and PstI (Thermo Scientific, USA) restriction sites in the case of protein Ec-1, SmtA and SpMTA or using Gibson Assembly Master Mix (New England Biolabs) for the rest of the proteins. The primer pair sequences used in the PCR reaction are provided in Supplementary Tables S4 and S4. The pTYB21 vectors encoding MT proteins were transformed into BL21(DE3)pLysS *E. coli* cells. All plasmids are deposited in Addgene under the following IDs: BcrMT1B (#200290), LIMT (#200291), SpMTA (#200292), XIMT (#200293), MacMT3 (#200294), OsMTI-1B (#200295), Ec-1 (#200296), PflQ2MT (#200297), SmtA (#200298), and TvMT (#200299). The culture medium (1.1% tryptone, 2.2% yeast extract, 0.45% glycerol, 1.3% K_2_HPO_4_, 0.38% KH_2_PO_4_) was prepared as described previously.^55,59^ Transformed bacteria (4 l) were cultured at 37°C until OD_600_ reached 0.7, then induced with 0.1 mM IPTG and incubated with 0.3 mM ZnCl_2_ overnight at 20°C with vigorous shaking. All subsequent steps of purification were conducted at 4°C. Cells were collected by centrifugation at 4000 ×*g* for 15 min, resuspended in 100 ml of cold buffer A (20 mM Na^+^-HEPES, pH 8.0, 500 mM NaCl, 1 mM TCEP) and sonicated three times for 15 min (5 s “on,” 10 s “off”) followed by centrifugation at 16 000 ×*g* for 45 min. The supernatant was incubated with 40 ml of chitin resin in buffer A and kept overnight with mild shaking. After the incubation resin was washed 10 times with 100 ml of buffer A, to induce the cleavage reaction 35 ml of buffer B (20 mM Na^+^-HEPES, pH 8.0, 500 mM NaCl, 100 mM DTT) was added to the resin and the mixture was incubated for 36–48 h at room temperature on a roller mixer. The centrifuged solution containing protein was subsequently concentrated to a small volume using Amicon Ultra-4 Centrifugal Filter Units with a membrane NMWCO of 3 kDa (Merck Millipore, USA) and then was acidified to pH 2.5 with 30% HCl. The protein was purified on a Superdex 75 Increase 10/300 column (Cytiva) in 10 mM HCl. The identity of apo-MT proteins was confirmed using an ESI–MS qTOF Bruker Compas mass spectrometer. Experimental and theoretical masses are presented in Supplementary Table S2. The DTNB assay was used for the determination of thiol concentration.^54^ Purified apo-MT proteins were used for experiments immediately after purification due to their instability under aerobic conditions.

### Reconstitution of zinc MTs

To obtain Zn(II)-loaded MT proteins aliquots of apo-MT forms in diluted HCl (pH 2.5 with 1 mM TCEP) under a nitrogen blanket were mixed with ZnSO_4_ depending on previously reported stoichiometries of zinc forms with 1.2–1.5 eq. excess.^81^ The pH of such solutions was adjusted to 8.6 with a 1 M solution of Tris base.^48^ MTs were concentrated with Amicon Ultra-4 Centrifugal Filter Units, containing a membrane cut-off of 3 kDa (Merck), and purified on Superdex 75 Increase 10/300 (Cytiva) in 20 mM Tris-HCl, pH 8.6. The concentrations of thiolates were determined using DTNB.^54^ Zn(II) concentration was analysed using PAR assay and ICP-OES iCAP 7400 Duo (Thermo Scientific). The samples were diluted in 10% HNO_3_ to confirm spectroscopic measurements.^58^

### CD

CD spectra of zinc MTs were recorded using a J-1500 Jasco spectropolarimeter (JASCO) at 25°C in a 2 mm quartz cuvette, under a constant nitrogen flow over the range of 190–300 nm with a 200 nm ⋅ min^−1^ speed scan. Final spectra were averaged from three independent scans, analysed using Jasco Spectra Manager software and presented as ellipticity θ in mdeg units. The concentration of MTs used for CD measurements was 20 µM in 10 mM Tris-HCl, 100 mM NaClO_4_, pH 7.4 with the addition of 360–400 µM TCEP.

### Electronic absorption spectroscopy in UV range

Electronic absorption spectra of thioneins and MTs were recorded using a JASCO V-650 spectrophotometer at 25°C in a 1 cm cuvette over the UV range of 200–300 nm. The pH-dependent measurements of 1 µM thioneins mixed with appropriate excess of Zn(II) in 100 mM NaClO_4_ solution were titrated with KOH in the pH range from 2.5 to 8. Metal-to-protein titrations were performed in 50 mM Borate buffer, pH 7.4 (0.1 M NaClO_4_) with 0.5 mM ZnSO_4_ or CdSO_4_ to a final metal-to-protein ratio of 7–18.^59^ TCEP was added as a weakly metal binding reducing agent to at least 5 molar equivalents excess over each cysteine thiol, and all titrations were performed under an argon atmosphere.^80^

### ESI–MS

ESI–MS experiments were performed on a quadrupole time-of-flight (qTOF) Bruker Maxis Impact mass spectrometer [Bruker Daltonik GmbH) calibrated with a commercial ESI–TOF tuning mix (Sigma-Aldrich]. Samples were directly infused with a 1 µl/min flow rate. ESI–MS spectra were recorded in positive mode with a capillary voltage of 2 kV, end plate offset potential of 500 V, nebulizer gas (N_2_) pressure of 1.5 bar, drying gas (N_2_) flow rate of 4 l/min, and drying temperature of 180°C. The mass range was set from 500 to 3000 m/z and recorded and averaged over 1 min. The Bruker Compass data analysis software package was used. Samples with apo-MTs were measured in solution water/methanol/formic acid (50/50/0.1). Complexes of holo-MTs with Zn(II) were prepared in 50 mM ammonium acetate, pH 7.4.

### Competition of MTs with ZnAF-2F fluorescent probe

Fluorescence measurements of the zinc probe ZnAF-2F were performed at 25°C with 492 nm excitation and 516 nm emission, with a Fluoromax (Horiba) spectrofluorometer in a single-use 1 cm × 1 cm fluorometric polystyrene cuvette. In the case of Zn(II) transfer study from MTs to ZnAF-2F, 0.5 µM MT in 50 mM Na^+^-HEPES buffer, pH 7.4 (0.1 M NaCl, 0.5 mM TCEP) was incubated with a zinc probe of various concentrations from 0.05 to 6 µM for 90 min and measured to obtain F values. Maximum (*F*_max_) and minimum (*F*_min_) probe fluorescence values were determined after addition of ZnSO_4_ or EDTA to their final concentrations of 75 and 250 µM, respectively.^47^ These values allow calibration of fluorescence response (*F*) and free Zn(II) concentration according to the formula (Eq. 1):


(1) \begin{equation*}{\left[ {{\mathrm{Zn}}\left( {{\mathrm{II}}} \right)} \right]}_{{\mathrm{free}}} = {K}_{\mathrm{d}} \cdot \frac{{\left( {F - {F}_{{\mathrm{min}}}} \right)}}{{\left( {{F}_{{\mathrm{max}}} - F} \right)}}\end{equation*}

In the case of Zn(II) competition to apo-MTs and ZnAF-2F, 0.5 µM apo-MT in 50 mM Na^+^-HEPES buffer pH 7.4 (0.1 M NaCl, 0.5 mM TCEP) was mixed with 2–4.5 µM ZnAF-2F and then titrated with increasing Zn(II) concentration from 0 to 8 µM. In practice the titration was performed in separate cuvettes to minimize titration time.^48^ All samples were incubated for 90 min and measured as earlier to reach *F, F*_min_, and *F*_max_ values necessary for free Zn(II) calculation. Dissociation constants of MTs were calculated using apparent *K*_d_ value of the Zn(II)-ZnAF-2F complex (5.5 nM) as provided in original work by Hirano *et al*.^83^

### Reaction of metal-free, partially and fully Zn(II)-loaded MTs with DTNB

Oxidation of metal-free, partially and fully Zn(II)-loaded MTs by DTNB was studied spectrophotometrically (JASCO V-650) in a kinetic mode at equimolar concentrations of the reactants (1 µM) at 25°C in 50 mM Na^+^-HEPES, pH 7.4 (0.1 M NaCl) at 412 nm. In some experiments weak and medium strength Zn(II) chelating agents (ATP, triphosphate, and NTA) were added to their final concentrations of 1 mM.^48,82^ Oxidation kinetics were monitored for 1–120 min depending on the protein and its metalation state. The data were fitted to the pseudo-first-order kinetic reaction in order to obtain *k*_obsd_.

### Competition of MTs with PAR

The chromophoric chelating probe PAR was used at high excess over MTs to study overall Zn(II) transfer from MTs. All experiments were performed in 50 mM Na^+^-HEPES buffer, pH 7.4 (0.1 M NaCl) treated with Chelex 100 (Bio-Rad). PAR and TCEP were added to a quartz cuvette containing the buffer to obtain a final concentration of 200 and 100 µM, respectively. The data were collected at 492 nm every 5 s. After 10 s from the start, MT was added to one of the cuvettes to a final concentration of 1 µM and the absorption was monitored for 60 min. Molar equivalents of Zn(II) transferred to PAR were calculated using the molar absorption coefficient of the ZnH*_x_*(PAR)_2_ complex at pH 7.4 equal to 71 500 M^−1^ cm^−1^. Dissociation constants of MTs were calculated using previously determined apparent dissociation constant of ZnH*_x_*(PAR)_2_ being *K*_d12_ = 7.1 × 10^−13^ M^2^ at pH 7.4.^58^

### Competition of bacterial MTs with complexones

The competition of selected MT with zinc chelators—common complexones—was determined spectropolarimetrically. For that purpose thioneins with a final concentration of 20 µM were incubated with 0.5 mM chelators with different Zn(II) fractional saturation from 5% to 85% for 24 h in 10 mM Tris-HCl buffer, 100 mM NaClO_4_, pH 7.4, in the presence of 360–400 µM TCEP. A metal-free sample was used as a control. Note that all cyclohexanediaminetetraacetic acid (CDTA), EDTA, and HEDTA chelators bind Zn(II) with 1:1 stoichiometry.^77^ The Zn(II) transfer was analysed by ellipticity measurements at 220, 242, 247 nm directly after mixing, then after 8 h. Ellipticity values recorded for various samples were then plotted as a function of initial free Zn(II) concentration, which was calculated based on known *K*_d_ values of the Zn(II) complex with a particular chelator.^81^ The data were fitted to the logarithmic version of Hill's equation as described previously.^76^ Based on obtained minimal and maximal ellipticity values, the fraction of Zn(II) transfer was calculated and used for the correction of free Zn(II) concentration values.

## Supplementary Material

mfad061_Supplemental_File

## Data Availability

The data underlying this article are available in the article and in its online supplementary material.
