# Supplementary material for: Differentiated Zn(II) binding affinities in animal, plant, and bacterial metallothioneins define their zinc buffering capacity at physiological pZn
Source: Metallomics. 2023 Oct 6;15(10):mfad061. doi: 10.1093/mtomcs/mfad061 (PMC10612145; doi:10.1093/mtomcs/mfad061)
Supplement: mfad061_Supplemental_File [file mfad061_supplemental_file.docx]

*Supplementary data*

Differentiated Zn(II) binding affinities in animal, plant, and bacterial metallothioneins define their zinc buffering capacity at physiological pZn

Karolina Szaro, Kinga Jurczak and Artur Krężel^*^

*Department of Chemical Biology, Faculty of Biotechnology, University of Wrocław, Joiot-Curie 14a , 50-383 Wrocław, Poland*

*Correspondence to: Artur Krężel, e-mail: artur.krezel@uwr.edu.pl

**Materials**

# Ethylenedinitrilotetraacetic acid (EDTA), hydroxyethylethylenediaminetriacetic acid (HEDTA), adenosine 5ʹ-triphosphate disodium salt (ATP), nitrilotriacetic acid trisodium salt monohydrate (NTA), 2-nitrophenylselenocyanate (NPSC) and 6-{2-[bis(2-pyridylmethyl)amino]ethylamino}-2ʹ,7'-difluorofluorescein) (ZnAF-2F) were purchased from Sigma-Aldrich. The metal-chelating resin Chelex 100 was from Bio-Rad. HCl, Tris base, dimethyl sulfoxide (DMSO), water (MS grade), formic acid LC-MS grade, and ammonium acetate were from VWR Chemicals. HCl (trace metal grade), ZnSO_4_·7H_2_O, ZnCl_2_, CdSO_4_ $\frac{\boldsymbol{8}}{\boldsymbol{3}}$ hydrate, 4-(2-pyridylazo)resorcinol (PAR), NaOH, boric acid, sodium tetraborate, pentasodium triphosphate, ammonium solution 25%, and *N*,*N*,*N′*,*N′*-tetrakis(2-pyridinylmethyl)-1,2-ethanediamine (TPEN) were obtained from Merck Millipore. 5,5ʹ-Dithiobis(2-nitrobenzoic acid) (DTNB) was from Tokyo Chemical Industry. NaClO_4_ was from Acros Organic B. V. B. A. Methanol LC-MS grade was from Witko (Łódź, Poland). Tris(2-carboxyethyl)phosphine hydrochloride (TCEP) was purchased from Iris Biotech GmbH (Marktredwitz, Germany). Tryptone, yeast extract, LB Broth, agar, isopropyl-β-ᴅ-1- thiogalactopyranoside (IPTG) and sodium dodecyl sulfate (SDS) were purchased from Lab Empire. Glycerol was from STANLAB (Lublin, Poland). Ampicillin, chloramphenicol, 1,4-dithiothreitol (DTT), 4-(2-hydroxyethyl)piperazine-1-ethanesulfonic acid sodium salt (HEPES) were from Roth. pTYB21 vector, Gibson Assembly Cloning Kit, restriction enzymes and chitin resin were from New England BioLabs. Aqueous solutions were configured with Milli-Q water (18.2 MΩ cm^−1^, 0.22 μm filter). Microporous membrane filters (0.22 and 0.45 μm) were used for further purification (Jet Biofil, China). All reagents were purchased from commercial suppliers and used accordingly. All buffers were prepared with Milli-Q water obtained with a deionizing water system (Merck). To eliminate trace metal ion contamination all pH buffers were treated with Chelex 100 resin and degassed over 2 h prior to use. The concentration of stock solutions of metal ion salts was 0.05 M, confirmed by representative series of ICP-MS measurements. For the culture of E. coli, Luria–Bertani (LB) medium and agar plates were used.

**Table S1**. Metallothioniens from various organisms used in this study.

| Domain or kingdom | Origin | Symbol | Amino acid sequence |
| --- | --- | --- | --- |
| *Animals* | *Callinectes sapidus* (Blue Crab) | BcrMT1B | MPGPCCNDKCVCQEGGCKAGCQCTSCRCSPCQKCTSGCKCATKEECSKTCTKPCSCCPK |
|  | *Littorina littorea* | LlMT | MSSVFGAGCTDVCKQTPCGCATSGCNCTDDCKCQSCKYGAGCTDTCKQTPCGCGSGCNCKEDCRCQSCSTACKCAAGSCKCGKGCTGPDSCKCDRSCSCK |
|  | Strongylocentrotus purpuratus (Purple Sea Urchin) | SpMTA | MPDVKCVCCTEGKECACFGQDCCVTGECCKDGTCCGICTNAACKCANGCKCGSGCSCTEGNCAC |
|  | *Xenopus laevis* | XlMT | MDPQDCKCETGASCSCGTTCSCSNCKCTSCKKSCCSCCPAECSKCSQGCHCEKGSKKCSCCN |
| Plants | Musa acuminata | MacMT3 | MSTCGNCDCVDKSQCVKKGNSYGIDIVETEKSYVDEVIVAAEAAEHDGKCKCGAACACTDCKCGN |
|  | *Oryza sativa japonica* | OsMTI-1B | MSCSCGSSCGCGSNCTCGKMYPDLEEKSSSAQATVVLGVAPEKAHFEAAAESGETAHGCGCGSSCKCNPCNC |
|  | *Triticum aestivum* | Ec-1 | MGCDDKCGCAVPCPGGTGCRCTSARSGAAAGEHTTCGCGEHCGCNPCACGREGTPSGRANRRANCSCGAACNCASCGSATA |
| *Bacteria* | *Pseudomonas fluorescens* | PflQ2MT | MNELRCGCPDCHCKVDPERVFNHDGEAYCSQACAEQHPNGEPCPAPDCHCERSGKVGGRDITNNQLDEALEETFPASDPISP |
|  | Synechococcus elongatus | SmtA | MTSTTLVKCACEPCLCNVDPSKAIDRNGLYYCSEACADGHTGGSKGCGHTGCNCHG |
|  | *Thermosynechococcus vulcanus* | TvMT | MTTVTQMKCACPHCLCIVSLNDAIMVDGKPYCSEVCANGTCKENSGCGHAGCGCGSA |

**Table S2**. Calculated and found monoisotopic masses of studied metallothioneins. Calculated masses were taken from Prot pi.^1^

| Metallothionein | calculated mass  of metal-free MT | found mass  of metal-free MT |
| --- | --- | --- |
| BcrMT1B | 6136.3629 | 6136.3546 |
| LlMT | 10031.7122 | 10030.6432 |
| SpMTA | 6383.2398 | 6383.1785 |
| XlMT | 6398.2467 | 6398.4342 |
| MacMT3 | 6768.8776 | 6767.8350 |
| OsMTI-1B | 7128.8181 | 7126.0031 |
| Ec-1 | 7706.9422 | 7704.8896 |
| PflQ2MT | 8937.8016 | 8928.0364 |
| SmtA | 5737.3832 | 5737.7390 |
| TvMT | 5770.3864 | 5770.4056 |

**Figure S1**. Deconvoluted mass spectra of investigated Zn(II)-loaded MTs obtained in this study. Spectra were recorded in 50 mM ammonium acetate.

**Table S3**. Calculated and found average masses of Zn(II) complexes of investigated metallothioneins recorded using ESI-MS. Calculated masses were taken from Prot pi.^1^

| Metallothionein | Species | Metallothionein  found mass | Metallothionein  calculated mass |
| --- | --- | --- | --- |
| BcrMT1B | Zn_6_MT  Zn_5_MT  Zn_4_MT | 6519.2976  6454.8564  6387.2772 | 6519.8247  6454.8247  6389.8247 |
| LlMT | Zn_9_MT  Zn_8_MT  Zn_7_MT  Zn_6_MT | 10606.4954  10540.5666  10475.4717  10408.4906 | 10608.2604  10543.2604  10478.2604  10413.2604 |
| SpMTA | Zn_7_MT  Zn_6_MT | 6830.2672  6765.1287 | 6830.6055  6765.6055 |
| XlMT | Zn_7_MT | 6844.4295 | 6845.8263 |
| MacMT3 | Zn_3_MT  Zn_2_MT | 6961.2552  6895.3205 | 6960.2754  6895.2854 |
| OsMTI-1B | Zn_4_MT  Zn_3_MT | 7384.0822  7319.1564 | 7383.4592  7318.4592 |
| Ec-1 | Zn_6_MT  Zn_5_MT | 8090.6019  8025.4466 | 8092.3976  8027.3976 |
| PflQ2MT | Zn_3_MT | 9132.7241 | 9132.8716 |
| SmtA | Zn_4_MT | 5993.4432 | 5994.3518 |
| TvMT | Zn_4_MT | 6026.7060 | 6027.5307 |

**Figure S2**. Spectrophotometric titrations of metal-free MTs with Cd(II) in UV range. Spectra were recorded for 1 µM apo-MT (blue lines) titrated with CdSO_4_. Cd(II)-saturated MTs are shown as red lines. Spectra were recorded in 50 mM borate buffer, 100 mM NaClO_4_, 45-135 µM TCEP, pH 7.4. Insets demonstrate absorbance increase at 240 (grey circles) and 260 nm (orange circles). Dashed lines indicate saturation of the signals.

**Figure S3**. Differential spectra of Zn(II) spectrophotometric titrations in UV range (**Figure 2**). Spectra were recorded for 1 µM apo-MT (blue lines) titrated with ZnSO_4_. Zn(II)-saturated MTs are shown as red lines. Spectra were recorded in 50 mM borate buffer, 100 mM NaClO_4_, 45-135 µM TCEP, pH 7.4.

**Figure S4**. Differential spectra of Cd(II) spectrophotometric titrations in UV range (**Figure S2**). Spectra were recorded for 1 µM apo-MT (blue lines) titrated with CdSO_4_. Cd(II)-saturated MTs are shown as red lines. Spectra were recorded in 50 mM borate buffer, 100 mM NaClO_4_, 45-135 µM TCEP, pH 7.4.

**Figure S5**. CD-monitored titrations of metal-free bacterial MTs with Cd(II). A) CD spectra of 20 µM apo-MT titrated with CdSO_4_ in 10 mM Tris-HCl, 100 mM NaClO_4_, 360 µM (SmtA) or 400 µM (TvMT) TCEP, pH 7.4. Red and blue lines show spectra of apo-MTs and Cd(II)-saturated MTs. Arrows indicate signal changes. B) Ellipticities at selected wavelengths as a function of increasing Cd(II)-to-apo-MT ratios.

**Figure S6.** Spectrophotometric pH titration of Zn(II)-MTs in 100 mM NaClO_4_. Red lines show Hill’s equation fits.

**Exemplary calculations of dissociation constants of Zn(II)-MT proteins used in this study with 4-(2-pyridylazo)resorcinol (PAR)**

*Calculation of K_d1_ of Zn_6_Ec-1 (Zn_6_MT):*

Dissociation of the weakest Zn(II) ion from Zn_6_Ec-1 occurs according to Eq. 1

Zn_6_MT + 2PAR ⇌ Zn_5_MT + Zn(PAR)_2_  (Eq. 1)

The concentration of Zn_6_MT was 1 µM, while PAR was 200 µM in 2 ml of Na^+^-HEPES buffer. Absorbance of Zn(PAR)_2_ complex measured at 492 nm in equilibrium state was 0.0383. Based on its molar absorption coefficient (71 500 M^-1^⋅cm^-1^)^2^ concentrations of reactants are:

[Zn(PAR)_2_] = 9.28⋅10^-7^ M (chromogenic Zn(II) complex)

[PAR] = 2⋅10^-4^ - 9.28⋅10^-7^ M = 1.9907⋅10^-4^ M (free PAR)

[Zn_6_MT] = Zn_6_MT_Total_ - [ZnH*_x_*(PAR)_2_] = 1.7⋅10^-6^ M - 5.36⋅10^-7^ M = 1.16⋅10^-6^ M

[Zn_5_MT] = Zn_6_MT_Total_ - [Zn_6_MT] = 5.36⋅10^-7^ M

The exchange constant *K*_ex1_ is described by Eq. 2

$K_{ex1}=\frac{[Zn\left( \mathrm{PAR} \right)_{2}]\cdot[\mathrm{Zn}_{5}\mathrm{MT}]}{\left[ \mathrm{Zn}_{6}\mathrm{MT} \right]\cdot{[\mathrm{PAR}]}^{2}}$ (Eq. 2)

The *K*_ex1_ value after substituting the concentrations of reactants is **6.19** **M^-1^**. Finally *K*_d1_ (the first dissociation constant) is calculated using Eq. 3 and the known dissociation constant of Zn(PAR)_2_, *K*_d12_^PAR^ = 7.1⋅10^-13^ M^2^.^2^

*K*_d1_ = $\frac{\left[ \mathrm{Zn}_{5}\mathrm{MT} \right]\left[ \mathrm{Zn}\left( \mathrm{II} \right) \right]_{\mathrm{free}}}{\left[ \mathrm{Zn}_{6}\mathrm{MT} \right]}=$*K*_ex1_⋅ *K*_d12_^PAR^ (Eq. 3)

*K*_d1_ = 6.19 M^-1^ **⋅** 7.1⋅10^-13^ M^2^ = 4.39⋅10^-12^ M

-log*K*_d1_ = **11.36**

*Calculation of K_d12_^av^ of Zn_7_XlMT (Zn_7_MT):*

Dissociation of two weakest Zn(II) ions from MT2 in exchange reaction with PAR occurs according to Eq. 4.

Zn_7_MT + 4PAR ⇌ Zn_5_MT + 2Zn(PAR)_2_ (Eq. 4)

The concentration of MT2 was 1 µM, while that of PAR was 200 µM in 2 ml of Na^+^-HEPES buffer. Absorbance of Zn(PAR)_2_ complex measured at 492 nm in equilibrium state was 0.1386. Based on its molar absorption coefficient (71 500 M^-1^⋅cm^-1^)^2^ concentrations of reactants are:

[Zn(PAR)_2_] = 1.94⋅10^-6^ M (chromogenic Zn(II) complex)

[PAR] = 2⋅10^-4^ - 1.94⋅10^-6^ M = 1.9806⋅10^-4^ M (free PAR)

[Zn_7_MT] = Zn_7_MT_Total_ - [Zn(PAR)_2_]/2 = 1.7⋅10^-6^ M - 9.69⋅10^-7^ M = 7.31⋅10^-7^ M

Dividing the [Zn(PAR)_2_] concentration by 2 results from Eq. 4.

[Zn_5_MT] = Zn_7_MT_Total_ - [Zn_7_MT] = 9.69⋅10^-7^ M

Exchange constant *K*_ex12_ is described by Eq. 5

$K_{ex12}=\frac{[\mathrm{Zn}_{5}MT2]{[Zn\left( \mathrm{PAR} \right)_{2}]}^{2}}{\left[ \mathrm{Zn}_{7}MT2 \right]\cdot{[\mathrm{PAR}]}^{4}}$ (Eq. 5)

The *K*_ex12_ value after substituting the concentrations of reactants is **3114** **M^-2^**. Finally *K*_d12_ (cumulative dissociation constant of the first two events) is calculated using Eq. 6 and the known dissociation constant of Zn(PAR)_2_, *K*_d12_^PAR^ = 7.1⋅10^-13^ M^2^.^2^

*K*_d12_ = $\frac{\left[ \mathrm{Zn}_{5}\mathrm{MT} \right]{[\mathrm{Zn}\left( \mathrm{II} \right)]}_{\mathrm{free}}^{2}}{\left[ \mathrm{Zn}_{7}\mathrm{MT} \right]}$=*K*_ex12_⋅ (*K*_d12_^PAR^)^2^ (Eq. 6)

*K*_d12_ = 3114 M^-2^ ⋅ (7.1⋅10^-13^ M^2^)^2^ = 1.56⋅10^-21^ M^2^

Since *K*_d12_ is a cumulative constant of two events and its direct comparison with *K*_d1_ is impossible, we used here *K*_d12_^av^, being the average value of *K*_d1_ and *K*_d2_. *K*_d12_^av^ is obtained by the square root of *K*_d12_ according to Eq. 7.

$K_{d12}^{av}=\sqrt{K_{d12}}$ (Eq. 7)

*K*_d12_^av^ = $\sqrt{1.56{10}^{-21} M^{2}}=3.95{10}^{-11}M$

*-*log*K*_d12_^av^ = **10.40**

*Calculation of K_d13_^av^ of Zn_9_LlMT (Zn_9_MT):*

Dissociation of three weakest Zn(II) ions from Zn_9_MT in exchange reaction with PAR occurs according to Eq. 8.

Zn_9_MT + 6PAR ⇌ Zn_6_MT + 3Zn(PAR)_2_ (Eq. 8)

Total concentration of Zn_9_MT was 1.0 µM, while that of PAR was 200 µM in 2 ml of Na^+^-HEPES buffer. Absorbance of Zn(PAR)_2_ complex measured at 492 nm in equilibrium state was 0.1386. Based on its molar absorption coefficient (71 500 M^-1^⋅cm^-1^)^2^ concentrations of reactants are:

[Zn(PAR)_2_] = 2.66⋅10^-6^ M (chromogenic Zn(II) complex)

[PAR] = 2⋅10^-4^ - 2.66⋅10^-6^ M = 1.9733⋅10^-4^ M (free PAR)

[Zn_9_MT] = Zn_9_MT_Total_ - [Zn(PAR)_2_]/3 = 1⋅10^-6^ M - 8.88⋅10^-7^ M = 1.12⋅10^-7^ M

Dividing the [Zn(PAR)_2_] concentration by 3 results from Eq. 8.

[Zn_6_MT2] = Zn_9_MT_Total_ - [Zn_9_MT2] = 8.88⋅10^-7^ M

Exchange constant *K*_ex12_ is described by Eq. 9

$K_{ex13}=\frac{[\mathrm{Zn}_{6}\mathrm{MT}]{[Zn\left( \mathrm{PAR} \right)_{2}]}^{3}}{\left[ \mathrm{Zn}_{9}\mathrm{MT} \right]\cdot{[\mathrm{PAR}]}^{6}}$ (Eq. 9)

The *K*_ex13_ value after substituting the concentrations of reactants is **2.55⋅10^6^** **M^-3^**. Finally *K*_d13_ (cumulative dissociation constant of the first three events) is calculated using Eq. 10 and the known dissociation constant of Zn(PAR)_2_, *K*_d12_^PAR^ = 7.1⋅10^-13^ M^2^.^2^

*K*_d13_ = $\frac{\left[ \mathrm{Zn}_{6}\mathrm{MT} \right]{[\mathrm{Zn}\left( \mathrm{II} \right)]}_{\mathrm{free}}^{3}}{\left[ \mathrm{Zn}_{9}\mathrm{MT} \right]}$=*K*_ex13_⋅ (*K*_d12_^PAR^)^3^ (Eq. 10)

*K*_d13_ = 2.55⋅10^6^ M^-3^⋅ (7.1⋅10^-13^ M^2^)^3^ = 9.05⋅10^-31^ M^3^

Since *K*_d13_ is a cumulative constant of three events and its direct comparison with *K*_d1_ is impossible, we used here *K*_d13_^av^, being the average value of *K*_d1_, *K*_d2_, and *K*_d3_. *K*_d13_^av^ is obtained by the cube root of *K*_d13_ according to Eq. 11.

$K_{d12}^{av}=\sqrt[3]{K_{d13}}$ (Eq. 11)

*K*_d13_^av^ =$\sqrt[3]{9.05\cdot{10}^{-31} M^{3}}$ $=9.67{10}^{-11}M$

*-*log*K*_d13_^av^ = **10.01**

**Figure S7**. Correlation of -log*K*_d_ values (-log*K*_d1_, -log*K*_d12_^av^ or -log*K*_d13_^av^ depending on MT) determined by PAR competition with -log*K*_d_ values (-log*K*_d1_, -log*K*_d12_^av^ or -log*K*_d13_^av^ depending on MT) determined by ZnAF-2F. The confidence level 95% is determined between blue lines.

**Figure S8**. Continuation from **Figure 8**. Titration of metal-free LIMT, SpMTA, OsMTI-1B, PflQ2MT, and SmtA with ZnSO_4_ in the presence of ZnAF-2F. A) 0.5 µM apo-MT and 2-4.5 µM ZnAF-2F (concentration varied depending on protein) in 50 mM Na^+^-HEPES,100 mM NaClO_4_, 500 µM TCEP. Orange line helps demonstrate titration end points. B) Semilog plots of the same data are presented to visualize changes during titration. Blue lines help to determine the number of tight zinc sites. C) Fluorescence responses were calibrated to obtain [Zn(II)]_free_ concentrations and are shown as a pZn (-log[Zn(II)]_free_) function of Zn(II)/apo-MT. Data are shown as means of n = 2 independent experiments + SD.

**Table S3**. Primer sequences for Gibson Assembly® Master Mix (New England Biolabs).

| Name of Primer | Primer sequence |
| --- | --- |
| pTYB21_F | 5’ GGTAATTAAATAACTAGTTGATCCG 3’ |
| pTYB21_ R | 5’ GTTCTGTACAACAACCTGAGATC 3’ |
| BcrMT1B_F | 5’ctcaggttgttgtacagaacATGCCGGGTCCGTGCTGT3’ |
| BcrMT1B_R | 5’caactagttatttaattaccTTATTTCGGACAGCAAGAGCACG3’ |
| LlMT_F | 5’ctcaggttgttgtacagaacATGTCCAGCGTGTTCGGC3’ |
| LlMT_R | 5’caactagttatttaattaccTTACTTGCAGGAACAAGAACGG3’ |
| XlMT_F | 5’ctcaggttgttgtacagaacATGGACCCGCAAGACTGTAAATG3’ |
| XlMT_R | 5’caactagttatttaattaccTTAGTTACAGCAGCTACATTTTTTGC3’ |
| MacMT3_F | 5’ctcaggttgttgtacagaacATGTCTACTTGTGGCAACTGTGATTG3’ |
| MacMT3_R | 5’caactagttatttaattaccTTAGTTGCCGCACTTGCAATC3’ |
| OsMTI-1B_F | 5’ctcaggttgttgtacagaacATGTCTTGCTCTTGCGGTTC3’ |
| OsMTI-1B_R | 5’caactagttatttaattaccTTAGCAGTTGCACGGGTTG3’ |
| TvMT_F | 5’ctcaggttgttgtacagaacATGACTACCGTTACCCAGATGAAATG3’ |
| TvMT_R | 5’caactagttatttaattaccTTATGCGGAACCGCAACC3’ |
| PflQ2MT_F | 5’ctcaggttgttgtacagaacATGAATGAACTGCGTTGC3’ |
| PflQ2MT_R | 5’caactagttatttaattaccTTACGGGGAGATTGGATC3’ |

**Table S4**. Primer sequences for Restriction Enzyme Cloning: SapI and PstI.

| Name of Primer | Primer sequence |
| --- | --- |
| Ec-1_F | 5’ GGTGGTTGCTCTTCCAAC 3’ |
| Ec-1_R | 5’ CTGCAGACCACC 3’ |
| SpMTA_F | 5’ GGTGGTTGCTCTTCCAAC 3’ |
| SpMTA_R | 5’ CTGCAGACCACC 3’ |
| SmtA_F | 5’ TTTTTTTGCTCTTCCAACATGACCTCGACGACCCTGGTG 3’ |
| SmtA_R | 5’ TTTTTTCTGCAGTTAACCGTGACAGTTGCAGCCCG 3’ |

**References**:

1. Prot pi, free-to-use bioinformatic tool: www.protpi.ch.

2. Kocyła A, Pomorski A, Krężel A. Molar absorption coefficients and stability constants of metal complexes of 4-(2-pyridylazo)resorcinol (PAR): Revisiting common chelating probe for the study of metalloproteins, *J Inorg Biochem* 2015;**152**:82–92.
